# Neutrophil extracellular traps in the central nervous system hinder bacterial clearance during pneumococcal meningitis

**DOI:** 10.1038/s41467-019-09040-0

**Published:** 2019-04-10

**Authors:** Tirthankar Mohanty, Jane Fisher, Anahita Bakochi, Ariane Neumann, José Francisco Pereira Cardoso, Christofer A. Q. Karlsson, Chiara Pavan, Iben Lundgaard, Bo Nilson, Peter Reinstrup, Johan Bonnevier, David Cederberg, Johan Malmström, Peter Bentzer, Adam Linder

**Affiliations:** 1 Division of Infection Medicine, Department of Clinical Sciences, Lund University, 22184 Lund, Sweden; 2 Center for Translational Neuromedicine, Blegdamsvej 3B, Copenhagen University, 2200 Copenhagen, Denmark; 3 Department of Experimental Medicine Science, Lund University, Solvegatan 19, 22184 Lund, Sweden; 4 Wallenberg Center for Molecular Medicine, Lund University, Solvegatan 19, 22184 Lund, Sweden; 5 Division of Medical Microbiology, Department of Laboratory Medicine, Lund University, 22184 Lund, Sweden; 6 Clinical Microbiology, Labmedicin, Region Skåne, 22184 Lund, Sweden; 7 Division of Anesthesia and Intensive Care, Department of Clinical Sciences, Lund University, 22184 Lund, Sweden; 8 Department of Neurosurgery, Lund University Hospital, 22185 Lund, Sweden; 9 Department of Anesthesia and Intensive Care, Helsingborg Hospital, 25187 Helsingborg, Sweden

## Abstract

Neutrophils are crucial mediators of host defense that are recruited to the central nervous system (CNS) in large numbers during acute bacterial meningitis caused by *Streptococcus pneumoniae*. Neutrophils release neutrophil extracellular traps (NETs) during infections to trap and kill bacteria. Intact NETs are fibrous structures composed of decondensed DNA and neutrophil-derived antimicrobial proteins. Here we show NETs in the cerebrospinal fluid (CSF) of patients with pneumococcal meningitis, and their absence in other forms of meningitis with neutrophil influx into the CSF caused by viruses, *Borrelia* and subarachnoid hemorrhage. In a rat model of meningitis, a clinical strain of pneumococci induced NET formation in the CSF. Disrupting NETs using DNase I significantly reduces bacterial load, demonstrating that NETs contribute to pneumococcal meningitis pathogenesis in vivo. We conclude that NETs in the CNS reduce bacterial clearance and degrading NETs using DNase I may have significant therapeutic implications.

## Abstract (web summary)

Neutrophils play critical roles in the host response to bacteria, including the production neutrophil extracellular traps (NET). Here the authors show that NET formation in the context of pneumococcal meningitis impairs bacterial clearance and targeting NET formation in this context could be a potential therapeutic option.

## Introduction

Acute bacterial meningitis (ABM) caused by *Streptococcus pneumoniae* (pneumococci) is a life-threatening medical condition that is accompanied by a high risk of debilitating neurological sequelae in survivors^1–3^. Pneumococci are not only one of the most frequent but are also one of the most lethal pathogens to cause ABM in both adults and children. Despite the introduction of antibiotics and vaccines, the mortality rates associated with pneumococcal ABM remains high^4,5^. The emergence of antibiotic-resistant strains and nonvaccine serotypes of pneumococci have further complicated therapy^6^.

The onset of bacterial infection results in the activation of both noncellular and cellular components of the immune system, with neutrophils among the first immune cells that are actively recruited to sites of infections^7^. The central nervous system (CNS) lacks extensive neutrophil immune-surveillance during healthy conditions, while during ABM a massive neutrophil recruitment occurs across the blood brain barrier (BBB) to eliminate bacteria^8^. In fact, the usually clear cerebrospinal fluid (CSF) takes on a turbid cloudy appearance due to the overwhelming presence of neutrophils and bacteria during pneumococcal ABM^9^.

The primary function of the neutrophils is to rapidly engage and clear invading pathogens. Neutrophils execute their function by phagocytosis, producing reactive oxygen species (ROS), hypochlorite and releasing antimicrobial protein/peptides through degranulation^7^. In addition, neutrophils have been described to resist invading bacteria by secreting decondensed nuclear DNA coated with granule-derived antimicrobial proteins called neutrophil extracellular traps (NETs) into the external environment^10^. NET formation is an evolutionarily conserved innate immune response that is directed at capturing and killing microbial pathogens^11^. The neutrophil-derived cationic proteins and proteases that coat NETs exhibit potent antimicrobial activity against a wide variety of microbial pathogens^12^. Recently, it was also reported that short DNA fragments derived from the DNA-backbone of NETs can also exert bactericidal effects against *Pseudomonas aeruginosa* and *Staphylococcus aureus*^13^. The role of NETs as an antibacterial strategy is only beginning to unravel and may be dependent on the type and site of infection^14–17^.

In spite of their antimicrobial properties, the excessive presence of NETs can be detrimental to the host in some cases, and hence NETs have been aptly acknowledged as “double-edged swords of innate immunity”^18^. NET-components such as extracellular DNA and antimicrobial proteins, including histones and neutrophil elastase (NE)^10^, are proinflammatory and contribute to disease severity^19,20^. Since NETs were discovered to have antimicrobial effects over a decade ago, several reports have demonstrated the prevalence of NET evasion mechanisms in bacteria, including pneumococci^21^. The pneumococci can evade NET-mediated killing by degrading NET-DNA secreting nucleases^22,23^, or by incorporating positively charged d-alanine into surface lipoteichoic acids, enabling the bacteria to repel cationic antimicrobial peptides bound to NETs^24^. These mechanisms allow pneumococci to remain trapped within NETs without being killed and aid in their dissemination from the lungs into the blood stream^21^.

To date, the presence of NETs has been demonstrated in the CSF in a single study by using a porcine model of *Streptococcus suis* meningitis^25^. However, the presence of NETs in human CSF has never been established. We undertook the study to investigate whether NETs are formed in CSF during pneumococcal infections and, if present, to characterize their functional importance in ABM. Here, we demonstrate the presence of NETs and NET-related proteins in the CSF of pneumococcal ABM patients using immunofluorescence microscopy and mass spectrometry. A rat model of pneumococcal meningitis further revealed neutrophil recruitment and NET formation in the CSF. The presence of NETs in the CSF compartment was found to promote pneumococcal survival and dissemination into other organs. Degradation of NET-associated DNA using DNase I resulted in the clearance of bacteria from the brain, lungs, spleen and blood of infected animals. This was because DNase treatment increased oxidative burst and phagocytosis in neutrophils coincubated with pneumococci. Our data indicate that NET formation in the CSF during pneumococcal meningitis might be detrimental to the host and that degrading these structures using DNase I leads to enhanced bacterial clearance from the brain.

## Results

### NETs are present in human CSF during pneumococcal meningitis

**Fig. 1:**
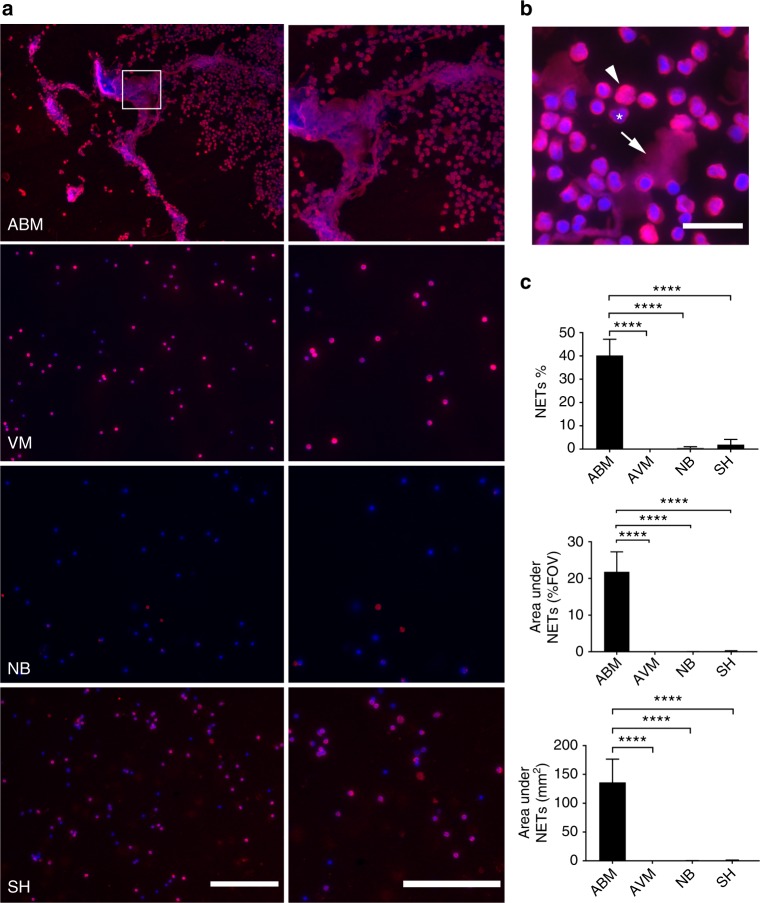
Neutrophil extracellular traps (NETs) are found in pneumococcal ABM patients. **a** NETs in human cerebrospinal fluid samples from patients with acute bacterial meningitis (ABM; *n* = 6), acute viral meningitis (VM; *n* = 4), neuroborreliosis (NB; *n* = 3), or subarachnoid hemorrhage (SH; *n* = 4) were visualized by immunofluorescence against human neutrophil elastase (red) and DNA (blue). Areas of red and blue colocalization represent NETs. **b** A close-up representative image of nonaggregated NETs with immunofluorescence against DNA (blue) and neutrophil elastase (red). Arrows indicate decondensed nonaggregated NETs, arrowheads indicate diffuse NETs with decondensed nuclei and * indicates neutrophils with intact nuclei. **c** NETs were quantified using Fiji and expressed as percentage of NETs, percentage of staining under NETs per field of view and total area under NET staining in square millimeters. All groups were compared to ABM by one-way ANOVA followed by Sidak’s multiple comparisons test, columns represent mean values,  ****P ≤ 0.0001 and error bars denote standard deviations. Scale bars denote 200 µm

**Fig. 2:**
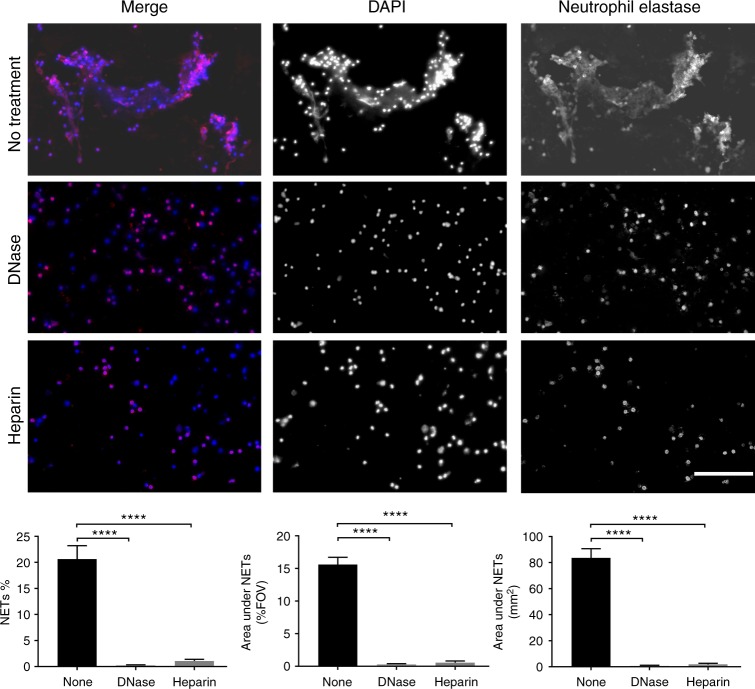
DNase and heparin degrade NETs ex vivo. Cerebrospinal fluid samples from some of the ABM patients (*n* = 3) were treated with DNase or heparin prior to visualization by immunofluorescence against neutrophil elastase (red) and DNA (blue). Areas of red and blue colocalization represent NETs. NETs were quantified using Fiji and expressed as percentage of NETs, percentage of staining under NETs per field of view and total area under NET staining in square millimeters. All groups were compared to ABM by one-way ANOVA followed by Sidak’s multiple comparisons test,  coulmns represent mean values, ****P ≤ 0.0001 and error bars denote standard deviations (n=3). Scale bars denote 200 µm

Apart from bacteria, NETs have been reported to be released in response to viruses^26^ and *Borrelia*^27^. The presence of neutrophils and cytokines can also cause damage to the CNS during sterile inflammation in subarachnoid hemorrhage (SH)^28,29^ These findings prompted us to investigate if NETs were present in human CSF during various infections and sterile noninfectious inflammation of the CNS. To examine the presence of NETs in CSF, cytospins from individuals afflicted with pneumococcal ABM, viral meningitis (VM), neuroborreliosis (NB) or SH were analyzed using immunofluorescence. The clinical parameters describing the patients are detailed in Supplementary Table 1a. Patients with ABM displayed distinctly higher neutrophil counts and protein content in the CSF compared to other disease groups. Areas of decondensed extracellular thread-like DNA or large DNA aggregates colocalizing with neutrophil-granule-derived NE, characteristic of NETs^30,31^, were detected by immunofluorescence exclusively in CSF from ABM patients but not in patients with VM, NB, and SH (Fig. 1a, c). Nonaggregated NETs and diffuse NETs^32^ with decondensed nuclei were also detected in ABM CSF at a higher magnification (Fig. 1b). Further, ex vivo treatment of CSF from ABM patients with previously validated NET-disruptive agents such as DNase I or heparin^33^ resulted in degradation of the NE-DNA complexes, confirming that these were NETs indeed (Fig. 2).

The extracellular release of NET-bound proteins such as heparin-binding protein (HBP/azurocidin)^34,35^, myeloperoxidase (MPO), NE^36^, and histones^37^ is tightly regulated as they can cause severe damage to host tissues. The substantial presence of these proteins in extracellular body fluids such as blood plasma is usually indicative of severe disease^20,38^. In fact, HBP is one such protein whose elevated levels in human plasma co-relate with sepsis severity^38,39^. Increased HBP concentrations are also observed in the CSF during ABM ^40^. To elucidate the presence of other NET-related proteins in the CSF environment, shotgun proteomic analysis was performed on the CSF of ABM, VM, NB, and SH patients. Significantly raised levels of several NET-associated antimicrobial proteins^34,41^ including MPO, NE, proteinase-3 (PR3), LL-37, matrix metalloprotease-9 (MMP-9), HBP, neutrophil gelatinase associated lipocalin (NGAL), S100A8/9 complex and histones were observed in the CSF from ABM patients compared to CSF from other patient groups (Table 1, Supplementary table 2).

**Table-1:** CSF of patients with acute bacterial meningitis (ABM), viral meningitis (VM), neuroborreliosis (NB) or subarachnoid hemorrhage (SH) was centrifuged to obtain soluble and pellet fractions

	

NET-associated proteins were detected by mass spectrometry in both fractions and compared in the form of a stacked histogram. The proteins were grouped according to localization within granules, cytosol or nucleus. Refer to Supplementary Table 2 included in the supplementary data for a detailed table containing all proteins discovered and statistics

Since NETs are known to be released in response to bacteria^12,42^, we tested if pathogens causing community-acquired or nosocomial ABM^43^ could trigger NET formation in vitro. Purified human neutrophils were challenged with clinical isolates of the meningeal pathogens *S. pneumoniae, Neisseria meningitides, Listeria monocytogenes, S. aureus, Escherichia coli, Acinetobacter baumanii, Streptococcus oralis, Staphylococcus capitis* and *Staphylococcus epidermidis* (Supplementary figure 2). All strains except *L. monocytogenes* significantly induced NET formation in vitro within an hour. This finding aligns with a previous report^44^ and is indicative that ABM-causing bacteria are the primary NET-inducers.

### NETs in the CNS promote pneumococcal survival in ABM

Having established the presence of NETs in patients with pneumococcal ABM, and pneumococci as a trigger for NET formation, we sought to explore the function of NETs during ABM in a rat model of pneumococcal meningitis. Four pneumococcal strains namely, SP001, SP002, SP003, and SP004, were isolated from patients with ABM where NET formation in the CSF was observed. The pneumococci were serotyped and characterized with data-dependent shotgun proteomics (Supplementary Table 3). Upon analyzing the proteomes, all clinical strains were found to express proteins that were highly similar to the well-documented pneumococcal strain TIGR4, indicating that the bacteria recovered from the ABM patients were indeed pneumococci (Supplementary Figure 2). The pneumococcal isolate SP001 (serotype 18b) induced maximal NET formation in purified human and rat neutrophils in vitro, and was therefore chosen for subsequent in vivo experiments (Supplementary Figs. 4, 6). Peptidyl arginine deiminase 4 (PAD4)-mediated histone citrullination facilitates chromatin decondensation and has been identified as a key event required for releasing NETs^45^. PAD4 inhibition using Cl-amidine results in the suppression of NET formation in response to bacteria^46^. Cl-amidine did not inhibit SP001-induced NET formation in purified human neutrophils, indicating that PAD4 is not required for NET production in response to SP001 (Supplementary Figure 5). Pneumococci are known to degrade NETs by secreting endonucleases such as EndA and TatD^22,23^. However, no degradation of NETs generated with SP001 was observed after 4 h of coincubation alone or in the presence of actin that is known to inhibit DNases^16^ (Supplementary figure 6). This is likely explained by the very low production of EndA and TatD in SP001 as shown in the mass spectrometry experiments (Supplementary dataset 1).

**Fig. 3:**
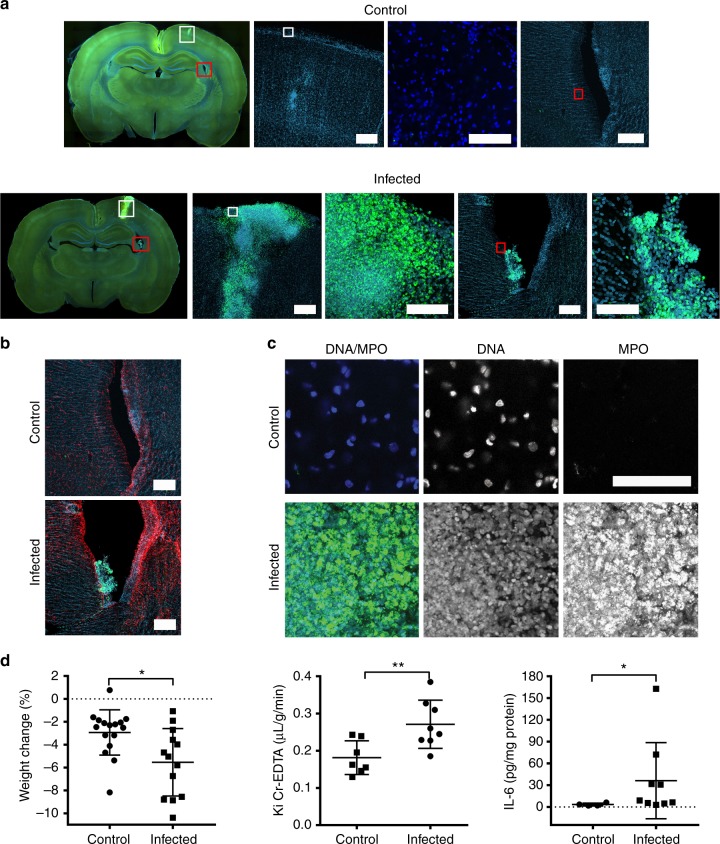
Pneumococci induce neutrophil influx and NET induction in the brain. Rats received a subarachnoid infusion of either *S. pneumoniae* SP001 strain (infected) or equal volume of saline solution (control). All animals were infected for 24 h and then sacrificed immediately. **a** Representative images from brain sections of rats injected with saline and clinical isolate SP001 using immunofluorescence against DNA (blue) and myeloperoxidase (green). White squares indicate parenchyma and red squares indicate right lateral ventricle. Scale bars denote 200 µm. **b** Representative images of right lateral ventricle in control and infected rats using immunofluorescence against DNA (blue), myeloperoxidase (green) and GFAP (red). Image 5 in the lower panel depicting infected brain sections is a closed-up of image 4 and depicts aggregated neutrophils in the right ventricle. Scale bars denote 200 µm. **c** Upon magnification of parenchymal regions, areas of colocalization of nuclear DNA with loss of distinct nuclear morphology (blue) and myeloperoxidase (green) indicated the presence of NETs. Scale bars denote 100 µm. **d** Rats were weighed prior to infection and again after 24 h postinfection to calculate the percent change in weight (n=13-16). Groups were compared by unpaired *t* test. Twenty-four hours after infection the blood to brain transfer constant (Ki) for ^51^Cr-EDTA, reflecting blood−brain-barrier permeability, was determined in infected and control animals (n=8-9). Groups were compared by unpaired *t* test. The rats were sacrificed, and the brain was removed and immediately homogenized. IL-6 was measured in the whole brain homogenates (n=4-9). Groups were compared by Mann−Whitney *U* test due to the non-normal distribution of the data, centre of line indicates mean values, *P ≤ 0.05, **P ≤ 0.01 and error bars denote standard deviations

**Fig. 4:**
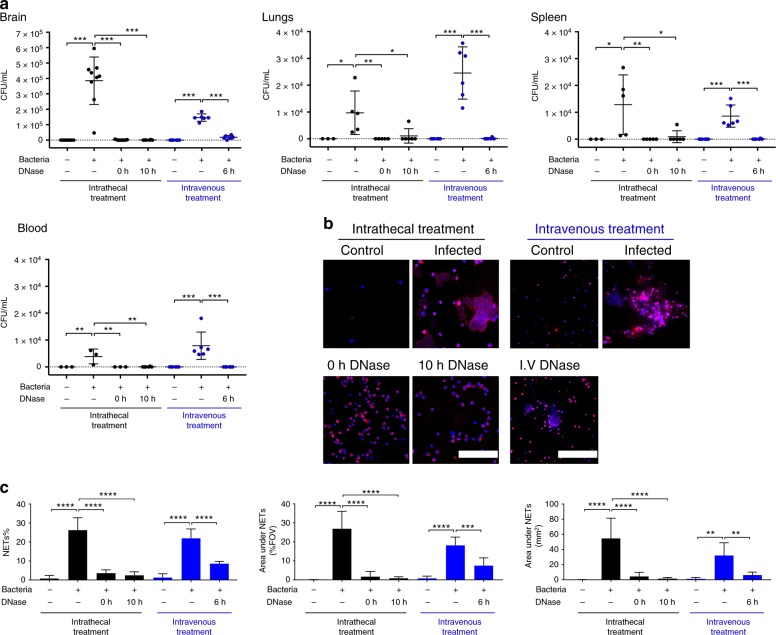
DNase facilitates bacterial clearance in a rat model of pneumococcal meningitis. Rats received a subarachnoid infusion of either *S. pneumoniae* SP001 strain (infected) or equal volume of saline solution (control). **a** After the rats were sacrificed, the brain, the right lung, and the spleen were collected and homogenized immediately. The blood was collected and centrifuged to obtain plasma. Organ homogenates and blood plasma samples were spread onto agar plates and resulting bacterial colonies were counted after 24 h. Indicated groups were compared by one-way ANOVA followed by Sidak’s multiple comparisons test, *P ≤ 0.05, **P ≤ 0.01, ***P ≤ 0.001 and error bars denote standard deviations. **b** To determine the effect of intrathecal DNase treatment, infected rats either received a subarachnoid infusion of 10 units of DNase simultaneously (0 h) or 10 h after the infection, or they received an equal volume of saline vehicle solution simultaneously to the infection. To determine the effect of intravenous DNase treatment, infected rats either received an intravenous bolus dose of 3500 units of DNase 6 h after the infection, followed by intravenous infusion of 780 units/h over the next 18 h, or they received an equal volume of saline vehicle control in the same manner. In all cases, uninfected (control) rats received an equal volume of saline vehicle control either intrathecally or intravenously as indicated. All rats were sacrificed 24 h after the infection. Cerebrospinal fluid was collected for visualization of NETs only by immunofluorescence against rat myeloperoxidase (red) and DNA (blue). Areas of red and blue colocalization represent NETs. Scale bars denote 200 µm. **c** NETs were quantified using Fiji and expressed as percentage of NETs, percentage of staining under NETs per field of view and total area under NET staining in square millimeters. Indicated groups were compared by one-way ANOVA followed by Sidak’s multiple comparisons test, centre line and columns indicate mean values **P ≤ 0.01, ***P ≤ 0.001, ****P ≤ 0.0001 and error bars denote standard deviations

Suspensions of pneumococci in saline or a saline vehicle control were infused subarachnoidally in rats. Immunofluorescence images of brain sections revealed the presence of MPO-positive neutrophils at the injection site and in the ventricles 24 h after infection (Fig. 3a). NETs were detected in the brain of infected rats in contrast to control animals that received a bolus of saline only (Fig. 3b). NETs appeared as smaller areas of decondensed extracellular DNA with bound MPO, and within large aggregates, characterized by substantial presence of DNA devoid of distinct nuclear morphology and MPO (Fig. 3b). An increase in the astrocyte activation marker glial fibrillary acidic protein (GFAP) staining, which is known to be upregulated in pneumococcal meningits^47^ was also observed in infected rats (Fig. 3c). Animals infected with SP001 lost more weight than controls (Fig. 3d). Meningitis in infected animals was validated at 24h by their increased BBB permeability using radiolabeled chromium-EDTA, higher IL-6 levels in the brain homogenates of infected animals compared to controls (Fig. 3d) and by the presence of viable bacteria in the whole brain homogenates of infected animals^48^ (Fig. 4a). The difference in weight loss between noninfected and infected animals was not significant as the surgical procedure alone can cause weight loss^49^ that often persists up to a week before full recovery is seen^50^.

Bacterial viability was determined by plating various dilutions of whole brain homogenates from control and infected animals. After 24 h, NETs could also be detected in the CSF of infected animals in amounts similar to those observed in human ABM (Fig. 4b, c). To clarify the role of NETs during ABM, DNase I (hereafter referred to as DNase) was infused subarachnoidally in rats either at the time of infection to prevent the accumulation of NETs, or 10 h after infection to simulate a postinfection treatment. No NETs were detected in the CSF of DNase-treated animals (Fig. 4b, c, Supplementary Figure 7). Pneumococcal viability in the brain homogenates of DNase-treated animals at both time points was also reduced significantly, indicating that DNase treatment had resolved the CNS infection (Fig. 4a). Since NETs can prevent bacterial dissemination from the site of infection^16,32^, bacterial load in other organs was investigated in DNase-treated animals. Surprisingly, lung and spleen homogenates, and blood plasma of DNase-treated animals displayed a reduction in viable bacteria compared to controls. Thus, indicating that DNase treatment also had prevented bacterial dissemination into other organs (Fig. 4a).

Given that intravenous injection is a more convenient and safer route of administration than intrathecal injection in a clinical setting, we also investigated effects of intravenous (i.v.) DNase treatment in the rat model in a randomized double-blinded manner. Intravenous DNase treatment also reduced bacterial loads in the homogenates of brain, lungs and spleen, and blood plasma (Fig. 4a). Intravenous DNase cleared NETs from the CSF (Fig. 4b, c, Supplementary Figure 7), suggesting that DNase can cross the BBB to facilitate bacterial clearance in this rat meningitis model. The bacterial clearance was also accompanied by lowered IL-1b levels in the brain homogenates of intrathecal and i.v. DNase-treated rats. This is indicative of a decrease in inflammation due to reduction in the number of viable bacteria in the brain (Supplementary Figure 8). A lower number of bacteria in the brain of untreated rats may have resulted due to infusion of intravenous saline and the use of heparin as an anticoagulant to prevent obstruction of catheters by blood clotting.

### DNase treatment enhances bacterial clearance

**Fig. 5:**
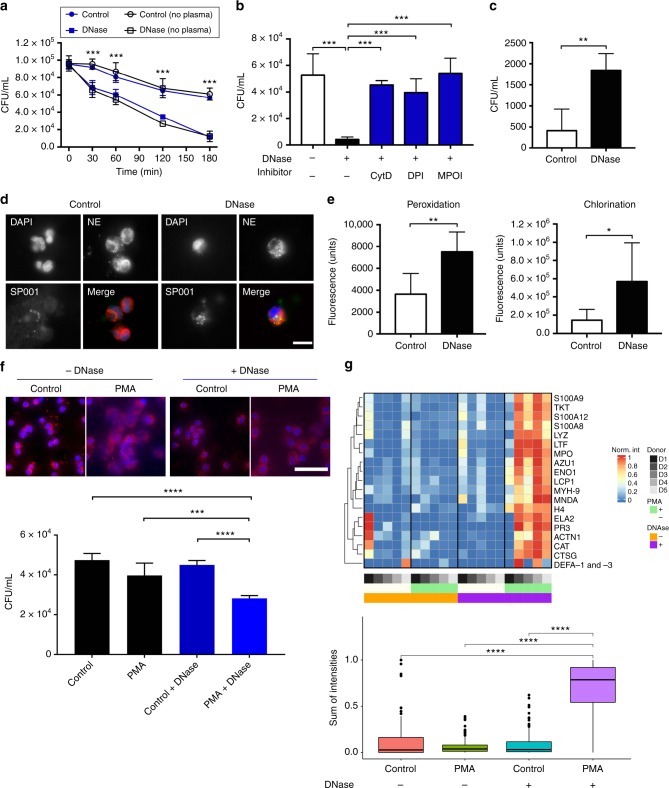
Mechanisms of DNase-mediated enhancement of bacterial killing. **a** Human neutrophils were stimulated with SP001 strain ± DNase and ±plasma. Time-dependent bacterial viability was determined (n=5). **b** Neutrophils were stimulated with the SP001 with/without DNase, in presence of saline, Cytochalasin D (CytD), diphenyleneiodonium (DPI) or 4-aminobenzoic acid hydrazide (MPOI) and bacterial viability was determined. Dots represent mean values and groups were compared by one-way ANOVA followed by Sidak’s multiple comparison test. **c** Neutrophils were stimulated with the SP001 ± DNase. Gentamicin was added and intracellular bacterial load was determined. Columns represent mean values and groups were compared by unpaired *t* test, n=5. **d** Neutrophils were stimulated with the Oregon Green-labeled SP001 (green). NETs were visualized by immunofluorescence against DNA (blue) and neutrophil elastase (red). Scale bars, 10 µm. **e** Peroxidation and chlorination due to MPO, in arbitrary fluorescence units, was measured in the supernatant after exposing neutrophils to SP001. Columns represent average values and groups  were compared by unpaired *t* test. Welch’s correction was applied to analysis of the chlorination graph because of unequal variance between the groups (F test), n=7. **f** Neutrophils stimulated with phorbol 12-myristate 13-acetate (PMA) with/without DNase were examined using immunofluorescence against DNA (blue) and neutrophil elastase (red). Scale bars, 50 µm. The untreated and DNase-treated supernatants were incubated with SP001 and bacterial viability was estimated. Sample supernatants from PMA + DNase treatments were compared to all other groups by one-way ANOVA followed by Sidak’s multiple comparison test, *P ≤ 0.05, **P ≤ 0.01, ***P ≤ 0.001, ****P ≤ 0.0001 and values are shown as means+  SD, n=5. **g** Supernatants were analyzed using DIA-mass spectrometry. Individual replicate protein intensities were normalized against respective total protein intensities per donor. Each row corresponds to an NET-bound protein with the gene name indicated on the right side. Each column represents a sample with the donor, PMA stimulation and/or DNase treatment indicated with annotations below the columns. Red−blue shades indicate the relative protein abundance of each sample. The data from the heat map are presented below as a boxplot with the individual replicates grouped as indicated on the *x*-axis and the relative NET-bound protein abundances are on the *y*-axis. Box boundaries represent first and third quartiles, centre line indicates median values. Whiskers extend to the extreme values (at most 1.5 times the interquartile range) and outliers are indicated individually as dots. Statistical analysis includes Kruskal–Wallis one-way analysis of variance and Wilcoxon rank sum test between groups; ****P < 0.001, n=5

To study the mechanisms by which DNase enhances neutrophil-mediated bacterial killing, NETs were induced in vitro by coincubating purified human neutrophils with pneumococci, in the presence of DNase or saline controls. Bacterial viability was reduced in DNase-treated samples within 30 min of stimulation, and maximal killing was observed after 3 h in vitro (Fig. 5a). Increased killing by neutrophils in the presence of DNase alone was also observed with several strains of antibiotic-resistant *S. pneumoniae* and methicillin-resistant *S. aureus* (Supplemental Figure 9). Bacteria coincubated with only DNase without neutrophils did not induce bacterial killing, suggesting that the DNase alone does not exhibit any antibacterial activity (Supplemental figure 10).

Phagocytosis followed by MPO-dependent hypochlorite production is a major neutrophil killing mechanism^51^, which may be hindered by the inability of the NET-bound neutrophils to reach NET-trapped bacteria. Hence, to determine the role of the phagocytosis-oxidative burst axis, NETs in vitro were induced by challenging isolated human neutrophils with pneumococci in the presence of DNase alone, or in conjugation with inhibitors of phagocytosis or oxidative burst. Inhibition of phagocytosis with cytochalasin D, and inhibition of either one of the two major enzymes in the oxidative burst (NADPH oxidase or MPO)^52^ significantly reduced bacterial killing (Fig. 5b). Compared to saline controls, a significant increase in phagocytosis was measured (Fig. 5c) and an increase in intracellular bacteria was observed within the neutrophils (Fig. 5d) 30 min after DNase treatment. MPO activity also significantly increased in the supernatant after DNase treatment after 30 min (Fig. 5e). Together these results suggest that removal of NETs by DNase enhances neutrophil killing mechanisms via the phagocytosis-oxidative burst axis.

**Fig. 6:**
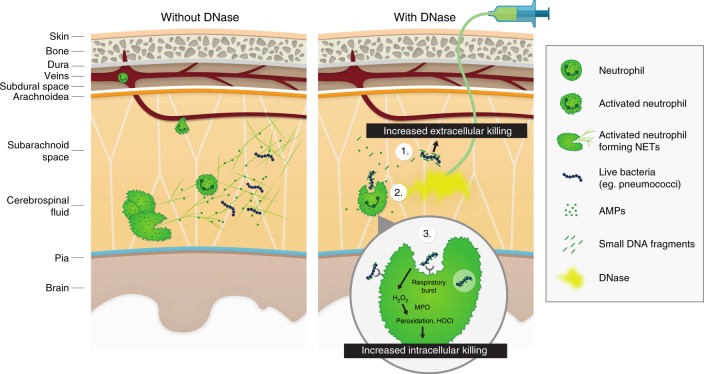
Model for activity of DNase in ABM. In ABM, in the absence of DNase (left panel), invasion of pneumococci into the CSF triggers neutrophil migration into the CNS resulting in pleocytosis and massive neutrophil extracellular trap (NET) formation. The trapped bacteria survive in the NETs because they are shielded from neutralization by cationic antimicrobial peptides and anionic extracellular DNA and are protected from phagocytosis by neutrophils. In the presence of DNase (right panel), the NETs are resolved due to degradation of extracellular DNA. (1) This generates antimicrobial DNA fragments and frees antimicrobial proteins, which exert bactericidal/bacteriostatic effects, leading to increased extracellular killing of bacteria. (2) The degradation of DNA also makes bacteria more accessible to intact neutrophils, leading to increased phagocytosis. (3) This increased contact with bacteria leads to activation of the respiratory burst pathway within the neutrophil, generating various peroxidated products and hypochlorite (HOCl), leading to increased intracellular killing of the bacteria

Extracellular DNA is a highly anionic polymer that can bind and neutralize activity of positively charged AMPs like LL-37 by forming complexes^53^. Degradation of DNA by DNase results in enhanced microbicidal activity due to an increase in freely available AMPs and unmasking of their positive charge. To determine the effects of DNase treatment that enhance AMP activity, cell-free supernatants from NETs generated with PMA in the presence or absence of DNase were collected and concentrated. A reduction in the viability of SP001 was observed upon incubation with concentrates derived from DNase-treated supernatants after 3 h in vitro (Fig. 5f). Mass spectrometry analysis revealed an increase in concentration of known NET-bound AMPs such as S100A8, S100A9, S100A12, lactoferrin, lysozyme, HBP/azurocidin, MPO, NE, histone H4, PR3, cathepsin G, alpha defensin-1 and -3 ^34^ in DNase-treated samples (Fig. 5g). Collectively, these results indicate that DNase degrades NETs at the site of infection, releasing functional antimicrobial peptides that boosts antimicrobial activity and enhancing phagocytosis-oxidative burst axis (Fig. 6).

## Discussion

During bacterial infections, NET formation in blood vessels is initiated by whole bacteria or bacterial products. Formation of NETs is mediated through a combination of CD11a/CD18 and TLR4 (toll-like receptor 4 ^54^, or CD11b/CD18 and TLR2 (toll-like receptor 2)^32^ signaling axes in neutrophils. The data describing the role of NETs in the CSF compartment during ABM are scarce. Our findings offer several novel insights into pneumococcal meningitis and uncovers a previously unrecognized contribution of NETs during CNS infections by pneumococci. NETs have been demonstrated to be beneficial in blood circulation and may have evolved as a mode of defense to immobilize and transfer microbes for later destruction in the reticuloendothelial system during systemic infections^55^. The lymphatic system in the CNS was first described by Foldi et al. in 1966 ^56^. The lymphatic vessels are present in the bed of the dura matter and drain into subarachnoid space. Recently, a detailed description of the lymphatic vessels lining the dura matter and immune-surveillance by T cells in them has been published^57^. However, the presence of neutrophils in lymphatic vessels of the CNS and whether these vessels carry neutrophils into the CNS during infections remains to be seen. Because the CNS has a poorly developed lymphatic drainage compared to most other organs^58^, overt NET formation could potentially hinder clearance of bacteria and waste products. Extracellular DNA can cause an increase in the viscoelasticity of airway secretions during cystic fibrosis^59^. A similar presence of extracellular DNA may also increase CSF viscosity and hinder its circulation in the CNS. Therefore, we hypothesize that excessive presence of NETs in the CSF compartment could be a major disadvantage for the host in ABM. Other important future areas of study will involve detailing the normal and pathological roles of NETs and the extracellular presence of NET-associated molecules such as HBP^60,61^ and histones^37^ in ABM.

Furthermore, our data demonstrate that targeting NET-associated extracellular DNA in the CNS with DNase administered either intrathecally or intravenously significantly increased bacterial clearance from the entire brain in a rat model of pneumococcal meningitis. These results align with a study from 1959 in which Tillett and coworkers suggested a 26% reduction in mortality by using DNase as an adjunct to penicillin therapy to treat pneumococcal meningitis^62^. The authors did not have the tools to provide a mechanism of action or the source of extracellular DNA at that time but postulated that degradation of DNA somehow exposed invasive bacteria to host defenses and/or antimicrobial therapy.

DNase treatment of neutrophils incubated with pneumococci resulted in reduced bacterial viability in a time-dependent fashion. The antibacterial effect exerted by neutrophils might be a complex interplay between phagocytosis, cationic peptide killing, ROS production, and NET formation. Phagocytosis is an important microbicidal mechanism that can also be observed in neutrophils post-NET formation^42^. Similarly, deficiencies in the NADPH oxidase and MPO are also associated with fatal infections^7^. Our data suggest that inhibition of phagocytosis, NADPH and MPO led to reduced bacterial killing with DNase treatment. Further, addition of DNase was paralleled by an increase in the intracellular bacterial load in neutrophils and MPO activity after 30 min. These data demonstrate the importance of classical neutrophil killing strategies such as phagocytosis and MPO activation in bacterial clearance even after NETs are rendered redundant by addition of DNase.

In instances where NET formation might dominate over other killing mechanisms, pneumococci that are known to resist NET-mediated killing^24^ may take advantage by hiding and replicating within the DNA framework. Thus, the NET-associated extracellular DNA that accumulates in the CNS could hinder the antimicrobial function of intact neutrophils and promote bacterial persistence. We have shown using in vitro studies that SP001 remains trapped in NETs as they do not produce DNases to degrade NETs. Taken together, these findings suggest that the bacteria remain trapped in the NETs without any reduction in viability. In the rat model, the bacteria escape from the brain and disseminate to other organs by unknown mechanisms. We hypothesize that bacteria upon encountering neutrophils induce NET formation in the brain; however, not all bacteria are bound to NETs. Hence, bacteria surviving within NETs or unbound bacteria increase their viable load and cause leakage in the BBB. The bacteria ultimately cross over the defective BBB and disseminate into other organs. Future studies delineating the exact mechanisms by which pneumococci escape the brain may be particularly important to understand pneumococcal pathogenicity.

The DNase treatment degrades extracellular NET-DNA and may facilitate phagocytosis by unmasking bacteria trapped in NETs, exposing them to intact neutrophils. In addition, we demonstrate that DNase treatment releases functional AMPs and theorize that DNase-mediated dissolution of NETs releases antimicrobials such as short DNA fragments^13,63^ and NET-bound cationic proteins that further increases bacterial killing in vivo. Thus, the overall effect of DNase may allow other neutrophil defense mechanisms to take over and facilitate bacterial clearance (Fig. 6).

Both intrathecal and intravenous DNase treatment results in the reduction of viable bacterial load across several organs, which can be considered beneficial^64,65^. However, we could not detect a significant effect on physiological parameters that indicate disease severity. We only show a reduction in the levels of IL-1b. The beneficial effects of DNase as an adjunct to antibiotics and/or anti-inflammatory agents such as dexamethasone in the treatment of bacterial meningitis needs to be further evaluated using relevant animal models. DNase is approved for the treatment of cystic fibrosis^66^, suggesting that clinical use of DNase as a novel therapeutic in ABM may be a feasible treatment option. Since DNase alone does not directly kill bacteria, the development of bacterial resistance to DNase treatment is less likely. In summary, our data suggest a harmful role of NETs in pneumococcal meningitis by inhibiting clearance, and reducing NETs using DNase I aids bacterial clearance in pneumococcal ABM.

## Methods

### Patient sample collection

The study was approved by the Lund University ethics committee (Dnr 2016/672) and consent was also obtained from patients or next of kin. CSF was obtained from patients with *S. pneumoniae* meningitis (*n* = 6), neuroborreliosis (*n* = 3), acute viral meningitis (*n* = 4), and subarachnoid hemorrhage (*n* = 3) by lumbar puncture or through an intraventricular device, if it was installed. CSF samples were kept at 4 °C and were processed within 0–24 h after collection. Antibiotic treatment was initiated in patients with pneumococcal ABM between 0 and 96 h from the time of CSF collection.

### Detection of NETs

CSF samples were fixed with 4% paraformaldehyde (Sigma-Aldrich). The samples were then cytocentrifuged (Thermo Fisher) and permeabilized and blocked with 5% goat serum (BioWest). ABM patients were also treated with either heparin (Leo, 1 U/mL), or DNase (Abcam, 5 U/mL). Human samples were stained with rabbit-anti-human neutrophil elastase (Dako, rabbit 1373 contract immunization, a kind gift from Dr Ole E. Sorensen, dilution 1:500) and detected with Alexafluor 594-labeled secondary goat-anti-rabbit F_ab_ antibody fragment (Life Technologies, dilution 1:1000). Rat samples were stained with anti-mouse MPO^67^ (Novusbio, NBP1-51148, dilution 1:500) and detected with Alexafluor 594-labeled secondary goat-anti-rabbit Fab antibody fragment (Life Technologies, dilution 1:1000). Coverslips (#1, Menzel Glaser) were mounted on top of the samples with ProLong Gold Antifade mountant (Life Technologies) and visualized with a Nikon Ti-E microscope. Images were acquired with the Andor Neo/Zyla camera and NIS elements advanced research software (Nikon).

### Quantification of NETs

For detection of NETs in patient CSF and animals, 5−7 random images at ×20 or ×10 magnification were used for quantifying each condition. Images were thresholded using auto local threshold function and Phansalkar or Otsu methods in Fiji software^68^. NETs were defined as a localization of DNA and neutrophil elastase and expressed as percentage of NETs, % NET staining per field of view and area under NETs staining in square millimeters.

NET formation in vitro was quantified as described by Mohanty et al.^17^. Five to seven random images at ×20 magnification containing a minimum of 175 cells were used for quantification. The DNA channel was used in identifying nuclei, and the elastase channel was determined using Fiji. The samples were then thresholded in Fiji and an increase in the elastase positive area of 33% was used to determine the NET-positive events.

### Bacterial culture

Clinical isolates of various bacteria were collected from either blood or CSF from patients after being diagnosed with meningitis at the clinic for infectious diseases at Skåne University Hospital in Lund, Sweden. For survival assays, animal infection and NET induction, all *S. pneumoniae* strains were cultured overnight on blood agar and then taken into Todd−Hewitt medium with 0.5% yeast extract (BD Biosciences) supplemented with 10% choline chloride (Sigma). Upon reaching an optical density (OD) of 0.4 they were washed and resuspended in phosphate-buffered saline (PBS) solution (Sigma) at an appropriate dilution for further experiments.

### Rat model of meningitis

The local Ethical Committee for Animal Research (M80-14) approved the experimental protocol^48^. Adult male Sprague Dawley® rats (Taconic, 350–370 g) were used. Animals were treated in accordance with the National Institutes of Health for the Care and Use for Laboratory Animals. Parasagittal craniotomy was performed (5 mm) using a drill without harming the dura. Either 20 µL of bacterial solution (3×10^6^ bacteria), or the same bacterial solution containing 10 units of recombinant human DNase I (Abcam), or sterile physiological saline solution (Fresenius Kabi), was injected into the subarachnoid space between the meninges through a polyethylene tube using a gas tight syringe. A needle (27 G) was inserted into the subarachnoid space and approximately 10 μL of CSF was drawn and diluted with 200 μL of physiological saline. NETs were visualized in CSF samples as above.

### Immunohistochemistry

After the animals were sacrificed, brains were quickly dissected and drop fixed overnight in 4% Paraformaldehyde (PFA) overnight (o/n) at 4 °C and washed in PBS. Brains were embedded in 1.5% agarose (A5093, Sigma-Aldrich) diluted in PBS and vibratome sectioned (100 µm sections; Leica VT1200S). Free floating sections were washed three times in PBS, blocked for 2 h at room temperature (RT) in blocking solution containing 0.3% Triton X-100 (Sigma-Aldrich) and 5% normal donkey or goat serum (Gibco™; Thermo Fisher Scientific) in PBS followed by overnight incubation at 4 °C with primary antibodies diluted in blocking solution. Primary antibodies used in this study were: chicken IgY anti-GFAP (1:500; PA1-10004, Thermo Fisher Scientific), mouse IgG1 anti-MPO (1:100; NBP1-51148, Novusbio) and rabbit anti-*S. pneumoniae* (SP; 1:100; PA1-7259, Thermo Fisher Scientific). The following day, sections were incubated with appropriate secondary antibodies conjugated to fluorophores (Alexa Fluor; 1:500; goat anti-mouse IgG1 AF568, goat anti-rabbit AF488, goat anti-rabbit AF568, goat anti-rat IgG2a AF488, goat anti-chicken AF568, Thermo Fisher Scientific) for 2 h at RT. After PBS washes, sections were counterstained with DAPI (4′,6-diamidino-2-phenylindole; 1:1000; 1 mg/mL; 62248, Thermo Fisher Scientific) for 10 min at RT. Slices were mounted with ProLong® Gold Antifade mounting medium (P36934, Thermo Fisher Scientific). Images of the immunolabeled slides were acquired using an epifluorescence microscope (Nikon Ni-E) with Plan Apo λ ×4 /0.20 objective at constant exposure levels throughout the study. Confocal microscopy analysis was also performed (Nikon Eclipse Ti) with Plan Fluor ×10/0.25 NA, ×20/0.75 NA, ×40/1.30 NA or ×100/1.25 NA oil objectives.

For immunohistochemistry analysis, maximum intensity projections were analyzed in ImageJ software and Imaris software (version 9.1.2).

### Mass spectrometry analysis

Proteins were reduced with 5 mM dithiothreitol (Sigma, USA) for 45 min at 37 °C, and alkylated with 25 mM iodoacetamide (Sigma, USA) for 45 min followed by dilution with 100 mM ammonium bicarbonate to a final urea concentration below 1.5 M. Proteins were digested by incubation with trypsin (1/100, w/w) for 18 h at 37 °C. The peptides were cleaned up by C18 reversed-phase spin columns as per the manufacturer’s instructions (Harvard Apparatus, USA).

All peptide analyses were performed on a Q Exactive Plus mass spectrometer (Thermo Fisher Scientific) connected to an EASY-nLC 1000 ultra-high-performance liquid chromatography system (Thermo Fisher Scientific). The DDA spectra were searched against the human reference proteome acquired from UniProt (UP000005640, Oct-2015, reviewed and canonical proteins only) by using Trans-Proteomic Pipeline (TPP v4.7 POLAR VORTEX rev 0, Build 201405161127) with X!Tandem. Computational workflows for analyzing DIA data was executed and managed by openBIS^69^ and included OpenSWATH v2.070 data extraction using an assay library of human purified neutrophils^70,71^ (unpublished: E. Malmström, S. Hauri, Johan Malmström), pyprophet-cli 0.0.1971 for false discovery rate (FDR) estimation and the FDR was set to 1% at peptide precursor level and at 1% at protein level, and TRIC^72^ for reducing the identification error. The quantitate protein matrix was filtered against NET-bound proteins^34^ and the individual replicate protein intensities were normalized against respective total protein intensity per donor. A more detailed description is described in the supplementary methods section.

### Blood−brain barrier permeability

BBB permeability was assessed by measuring the blood to brain transfer constant (Ki) for ^51^Cr-EDTA as described in detail previously^73^. Briefly, at about 23 h after injection of bacteria, animals were reanesthetized. A bolus of approximately 50 kBq of ^51^Cr-EDTA (Nycomed Amersham, Stockholm, Sweden) was given intravenously, followed by an infusion at a rate of 200 kBq/h. Arterial blood samples (10 µL) were collected at every 2.5−5 min for 40 min. At 37 min, about 70 kBq ^125^I-albumin was given intravenously. At 40 min the animals were decapitated. An 8-mm coronal section of the cortex centered over the insertion point for bacteria was removed and weighed. ^51^Cr and ^125^I activities in tissue and blood samples were determined in a gamma counter. Arterial hematocrit was measured at the start and at the end of the tracer infusion and was used to convert blood concentrations into plasma concentrations. Ki was then calculated as: Ki = *B*/∫_0_^*T*^ Ca (*t*) dt, where *B* is the amount of tracer in the tissue, Ca is the concentration of the tracer in arterial plasma as a function of time, and *T* is the duration of the experiment. *B* was calculated as the total ^51^Cr activity in the tissue samples minus the ^51^Cr activity in the cortical plasma volume determined by the distribution volume for ^125^I-albumin in the tissue sample.

### 10-h DNase treatment in rats

Recombinant human DNase I (Abcam) was infused subarachnoidally 10 h after bacterial infection. A catheter was placed subarachnoidally as described above and 10 units of DNase was infused at a flow rate of 2 μL/min. The piece of bone was replaced and sealed with histoacryl and the wound was again closed.

### Intravenous DNase treatment in rats

All intravenous treatments were performed in a blinded and randomized fashion. The correspondence between code and treatment was revealed only after results were obtained. Bacteria or saline vehicle control was infused subarachnoidally in rats as described above. For intravenous administration, a catheter was inserted into the jugular vein. Six hours after bacteria or vehicle infusion, a bolus dose of 3500 units of DNase (Worthington) or equal volume saline vehicle solution was infused intravenously using a syringe pump. Then a continuous infusion of 780 units/h of DNase or equal volume saline vehicle solution at a flow rate of 0.05 mL/h was initiated.

### Bacterial counts in rat organ homogenates

The brain, lungs and spleen were homogenized using silicone beads in a TissueLyser (Qiagen) and the homogenate was plated onto blood agar plates and incubated at 37 °C overnight. Colony-forming units were counted.

### IL-6 in brain homogenate

Brain homogenate was mixed with T-PER lysis buffer (Thermo Fisher) according to the manufacturer’s directions. Protein content in the lysate was measured by bicinchoninic acid (BCA) protein assay kit (Thermo Fisher). IL-6 was measured in the lysed homogenate using a Quantikine rat IL-6 ELISA kit (R&D Systems) according to the manufacturer’s directions.

### Bacterial killing after DNase treatment

The leakage of plasma across BBB further increases the plasma protein concentrations in the CSF in ABM, which can vary between 500 and 3600 mg/dL^74^. Hence, 10% plasma (700 mg/dL) diluted in Hank's balanced salt solution (HBSS) buffer, representing the lower limit was chosen to mimic the protein concentration of ABM CSF. NETs were induced in human neutrophils as above, in the presence of DNase (5 units) or equal volume of saline solution. To determine the killing mechanism, the following inhibitors were added 30 min after addition of DNase and bacteria: 10 µM Cytochalasin D (Calbiochem), 10 µM myeloperoxidase inhibitor 4-Aminobenzoic hydrazide (4-ABAH) (Calbiochem) or 10 µM NADPH oxidase inhibitor diphenyl iodonium chloride (DPI) (Calbiochem). Samples were incubated for 2.5 h after addition of inhibitors. NETs were detected as above using immunofluorescence. Samples were then diluted and plated on blood agar plates. Colony-forming units of *S. pneumoniae* were determined.

### Myeloperoxidase (MPO) activity assay

After 30 min of in vitro NET induction in the presence and absence of DNase as described above, samples were centrifuged, and the cell-free supernatant analyzed for MPO peroxidation and chlorination activity using EnzChek MPO Assay Kit (Molecular Probes) according to the manufacturer’s directions.

### Gentamicin assay

SP001 was cultured in THY medium supplemented with choline chloride as described above. The bacteria were then added at MOI of 10 to 1×10^6^ neutrophils in HBSS with 10% plasma alone, or in the presence of DNase (5 U) for 15 min. Gentamicin (10 µg/mL) was added for 30 min at 37° to kill extracellular bacteria. Cells were washed thrice with HBSS and lysed with sterile water. The bacteria were then plated out at various dilutions and CFUs were counted to assess survival.

### Visualization of phagocytosis

SP001 strain was labeled with Oregon green 488-X succinimidyl ester (20 µM, Life Technologies) in PBS for 30 min in the dark at room temperature. The labeled bacteria were then washed thrice in PBS to remove excess unbound dye. Bacteria were then added to neutrophils in HBSS with 10% autologous plasma (MOI 1:10) alone or in the presence of DNase (5 U) on shaking for 1 h at 37° in the dark. Samples were then fixed, cytocentrifuged onto slides and processed for immunocytochemistry as described previously. Neutrophils were probed with rabbit human anti-elastase and DNA was visualized with DAPI.

### Antibacterial assays using cell-free supernatants

NETs were generated with 1×10^6^ purified human neutrophils using PMA (Sigma-Aldrich) in low-bind Eppendorf tubes. NETs were treated with 5 U DNase for 20 min for the complete dissolution of NETs and the supernatants were collected. Samples were then concentrated using 10 K centrifuge filters (Microcon, Millipore). SP001 were grown to mid log phase. 10 µL of the concentrate was incubated with 90 µL of 2×10^6^ CFU/mL of SP001 for 3 h at 37 °C in incubation buffer (10 mM Tris and 140 mM NaCl; pH = 7.4). This was followed by plating of serial dilutions on blood agar plates and overnight incubation at 37 °C and CFUs were enumerated after 24 h. For MS analysis, protein estimation was performed using the BCA kit (Pierce). Twenty micrograms of protein was taken for digestion and peptide clean-up as described above.

### Statistical analysis

All statistical analyses were performed with R version 3.2.3 and GraphPad Prism 7. One-way ANOVA with Sidak’s multiple comparisons test was used for all comparisons between groups, except for comparison between groups for assessment of differences in weight loss, BBB permeability and IL-6 where Mann−Whitney *U* test was used; and for differences in the sum intensities of spectra, where Kruskal–Wallis one-way analysis of variance and Wilcoxon rank sum test between groups were used.

### Reporting summary

Further information on experimental design is available in the Nature Research Reporting Summary linked to this article.

## Supplementary information

Supplementary Information

Description of Additional Supplementary Files

Supplementary Data 1

Reporting Summary

## Data Availability

MS data have been deposited to ProteomeXchange via MassIVE partner repository with accession codes PXD011546. All other data supporting the findings will be made available upon reasonable request to the corresponding author.
