# Supplementary material for: Neutrophil extracellular traps in the central nervous system hinder bacterial clearance during pneumococcal meningitis
Source: Nat Commun. 2019 Apr 10;10:1667. doi: 10.1038/s41467-019-09040-0 (PMC6458182; doi:10.1038/s41467-019-09040-0)
Supplement: Supplementary file 1 — Supplementary Information [file 41467_2019_9040_MOESM1_ESM.pdf]

Supplementary information for “Neutrophil extracellular traps in the central nervous system hinder bacterial clearance during pneumococcal meningitis” by Mohanty et. al.

Includes:

Supplementary tables 1-3

Supplementary figures 1-13

Supplementary methods

Supplementary references

Supplemental Figures

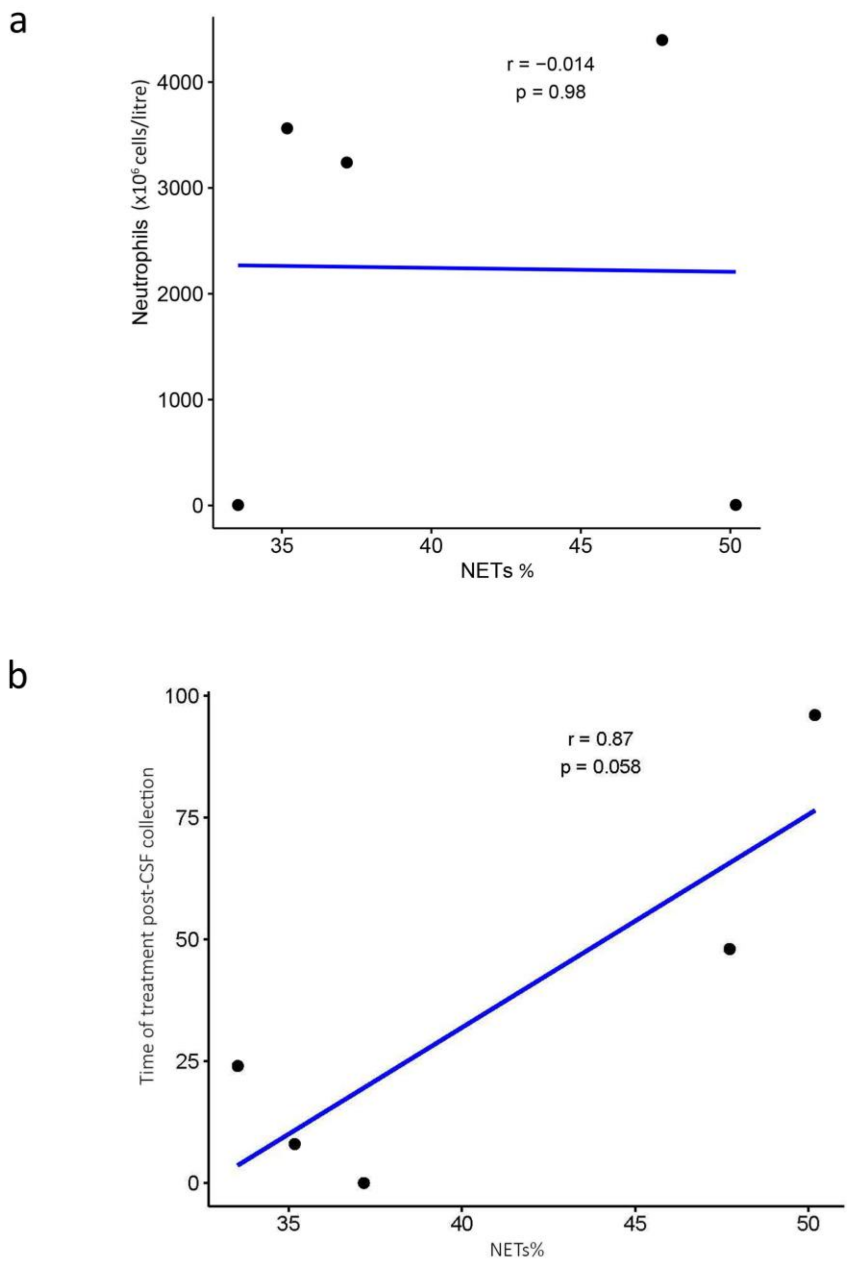

Supplementary figure 1 related to figure 1. a Linear regression analysis of cell number and NETs% in ABM patients. b Linear regression analysis of time of treatment post-CSF collection and NETs%.

Supplementary table 1. Related to Figure 1 – Patient characteristics

	**ABM (n=6)**	**VM (n=3)**	**NB (n=3)**	**SH (n=4)**
**Male (n, %)**	3, 50%	1, 33%	2, 66%	1 (25%)
**Age** ***(mean ± SD)***	46 ± 24	29 ± 13	57 ± 10	48 ± 23
**Pathogen (n, %)**	*S. pneumoniae* (6, 100%)	Enterovirus (2, 66%) UNS, 1 (33%)	*B. burgdorferi*, (3, 100%)	None, (4, 100%)
**Identification method**				
Blood culture (n, %)	6, 100%	-	-	-
CSF culture (n, %)	1, 17%	-	-	-
CSF PCR (n, %)	5, 83%	2, 66%	-	-
Serology (n, %)	**		3, 100%	-
Laboratory variables in CSF (mean ± SD)				
Neutrophils ($x10^6$/L)	2240 ± 2090	61 ± 47	7 ± 13	283 ± 324
Monocytes ($x10^6$/L)	211 ± 233	154 ± 148	121 ± 151	374 ± 619
Erythrocytes ($x10^6$/L)	475 ± 585	<300	<300	141000 ± 130000
Lactate (mmol/L)	7.0 ± 4.4	2.3 ± 0.6	2.2 ± 0.6	4.0 ± 1.1
Glucose (mmol/L)	5.3 ± 3.2	4.7 ± 1.4	3.4 ± 0.1	4.3 ± 0.8
Albumin (g/L)	824 ± 1167	394 ± 87	640 ± 267	759 ± 712
Protein (g/L)	2.3 ± 2.4	0.59 ± 0.11	0.97 ± 0.40	1.58 ± 1.45

****** *See supplementary table 3 for further details.*

ABM = Acute bacterial meningitis, VM = Viral meningitis, NB = Neuroborreliosis

SH = Subarachnoid hemorrhage

Supplementary table 2. Related to Figure 1. – Spectral counts of NET-associated proteins in CSF

		ABM (n = 6)		NB (n = 3)		VM (n = 3)		SH (n = 4)	
		Mean	SD	Mean	SD	Mean	SD	Mean	SD
**Pellet**	**MPO**	49.0	34.9	0.0**	0.0	5.3*	7.6	4.3**	8.5
	**NE**	13.0	5.3	0.0**	0.0	1.0**	1.0	1.3**	1.9
	**PR3**	7.3	2.3	0.3**	0.6	1.7**	2.1	1.3**	1.5
	**LL-37**	9.7	2.1	0.0**	0.0	0.3**	0.6	0.5**	1.0
	**MMP-9**	17.7	3.2	0.0**	0.0	2.0*	3.5	0.8**	1.5
	**HBP**	9.0	7.2	0.0*	0.0	0.0	0.0	0.5*	1.0
	**NGAL**	16.7	9.9	0.3**	0.6	2.0*	3.5	2.0**	3.4
	**S100A8**	34.0	26.9	5.3	6.7	5.7	4.9	5.3	3.4
	**S100A9**	119.7	17.8	9.7**	12.5	11.3**	11.0	7.8**	7.9
	**H1.2**	1.0	1.0	0.0	0.0	1.7	2.1	0.0	0.0
	**H1.4**	1.7	1.5	0.0	0.0	0.3	0.6	0.0	0.0
	**H4**	14.7	16.7	2.7	2.5	8.0	6.6	4.0	1.4
**Soluble**	**MPO**	14.7	3.2	0.0	0.0	2.7	1.2	0.7	1.2
	**NE**	5.0	3.6	0.0	0.0	1.3	0.6	0.0	0.0
	**PR3**	2.0	1.0	0.3	0.6	1.7	0.6	0.3	0.6
	**LL-37**	5.7	3.8	0.3*	0.6	0.3*	0.6	0.3*	0.6
	**MMP-9**	19.3	12.1	0.0**	0.0	0.0**	0.0	0.0**	0.0
	**HBP**	2.7	2.1	0.0	0.0	0.0	0.0	0.0	0.0
	**NGAL**	14.0	4.0	1.0*	1.7	1.0*	1.0	0.0*	0.0
	**S100A8**	12.7	4.2	1.0	1.7	3.7	3.8	1.7	2.1
	**S100A9**	20.7	13.7	2.3	4.0	4.0	6.9	3.0	4.4
	**H1.2**	0.7	0.6	0.0	0.0	0.3	0.6	0.0	0.0
	**H1.4**	1.0	1.0	0.3	0.6	0.0	0.0	0.0	0.0
	**H4**	4.0	3.6	2.0	2.6	2.7	0.6	1.0	1.7

ABM = Acute bacterial meningitis, VM = Viral meningitis, NB = Neuroborreliosis

SH = Subarachnoid hemorrhage

***p<0.05, **p<0.01 compared to ABM by one-way ANOVA followed by Sidak's multiple comparison test to adjust for the number of comparisons in each group and Bonferroni-adjusted for the number of proteins analyzed.**

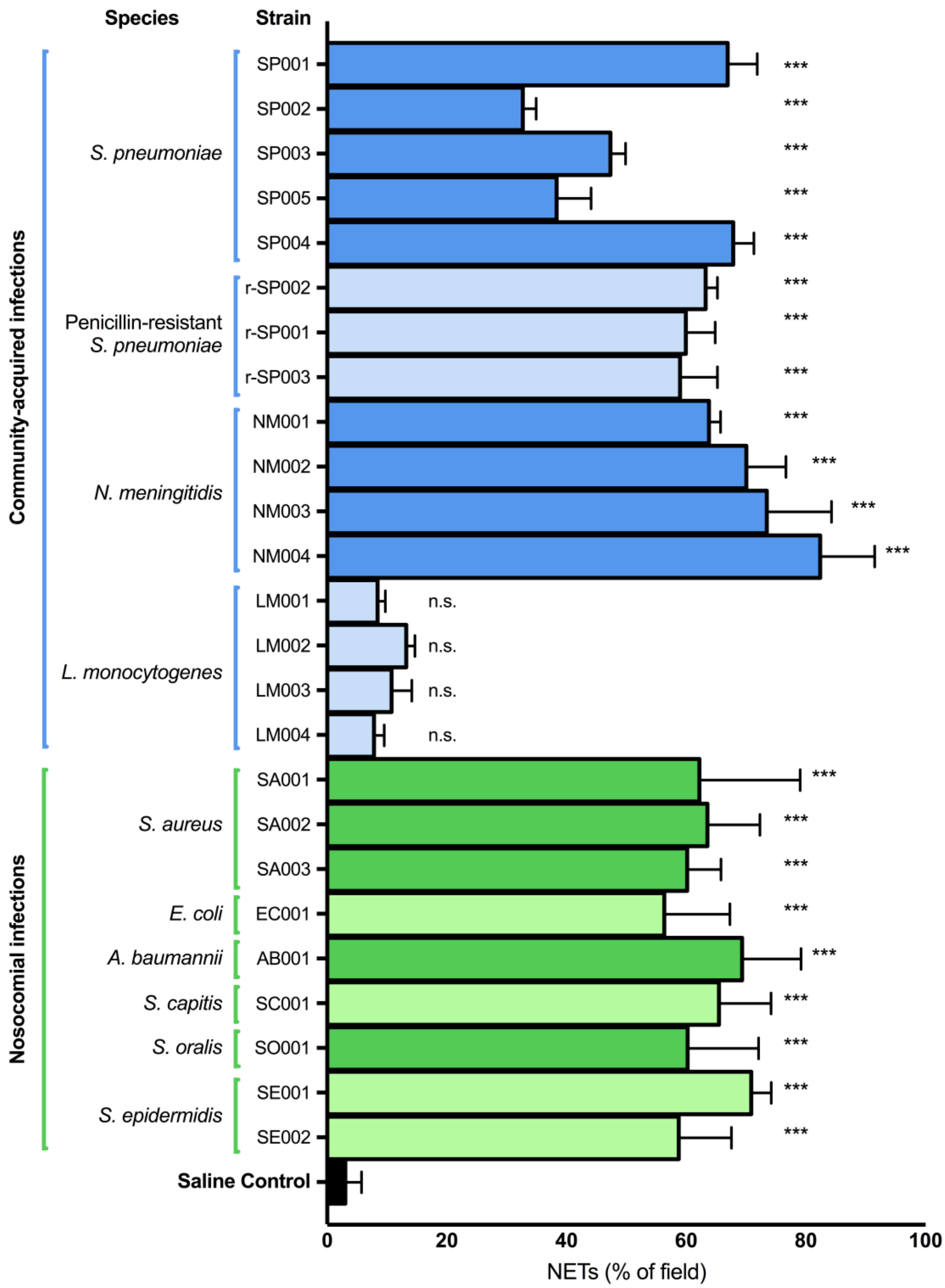

Supplementary figure 2. Related to Figure 2. NETs are formed in vitro by bacteria isolated from patients with acute bacterial meningitis Neutrophils were stimulated with several bacterial strains isolated from either the CSF of blood of patients with acute bacterial meningitis. After 3 hours, NETs were visualized by

immunofluorescence against human neutrophil elastase and DNA. The amount of NETs as a percentage of the total area was determined using Fiji. All groups were compared to the saline control group by one-way ANOVA followed by Sidak's multiple comparisons test (*** $P \leq 0.001$, n.s. not significant). Bars denote mean + standard deviation.

Supplementary table 3. Related to Figure 2. – Characterization of clinical strains of pneumococcal.

	SP001	SP002	SP003	SP004	TIGR4
**Total proteins detected**	996	990	1047	917	1104
**Total peptides detected**	12942	12654	12852	9955	13628
**Total proteins detected with at least 5 scans**	594	589	624	500	638
**Number of proteins with Log_2 Fold change of 2 up regulation**	2	4	7	4	_
**Number of proteins with Log_2 Fold change of -1 down regulation**	13	25	8	28	_
**Strain Serotype**	18b	8	11a	3	4

Clinical isolates of NET-inducing pneumococci, namely SP001, SP002, SP003 and SP004, isolated from patients suffering ABM where NETosis was also observed, were cultured under similar growth conditions along with the TIGR4 strain. TIGR4 was used as the reference pneumococcal strain to which all other clinical isolates were compared. Data-dependent (DDA) shotgun proteomics was then performed on the bacterial cultures. Spectral counts were extracted from the raw data by searching against proteome library of the TIGR4 strain (Swiss-Uniprot ID -

UP000000585) using X! Tandem. The details of the data obtained from the shotgun proteomics and the serotypes of the strains are included in the table above.

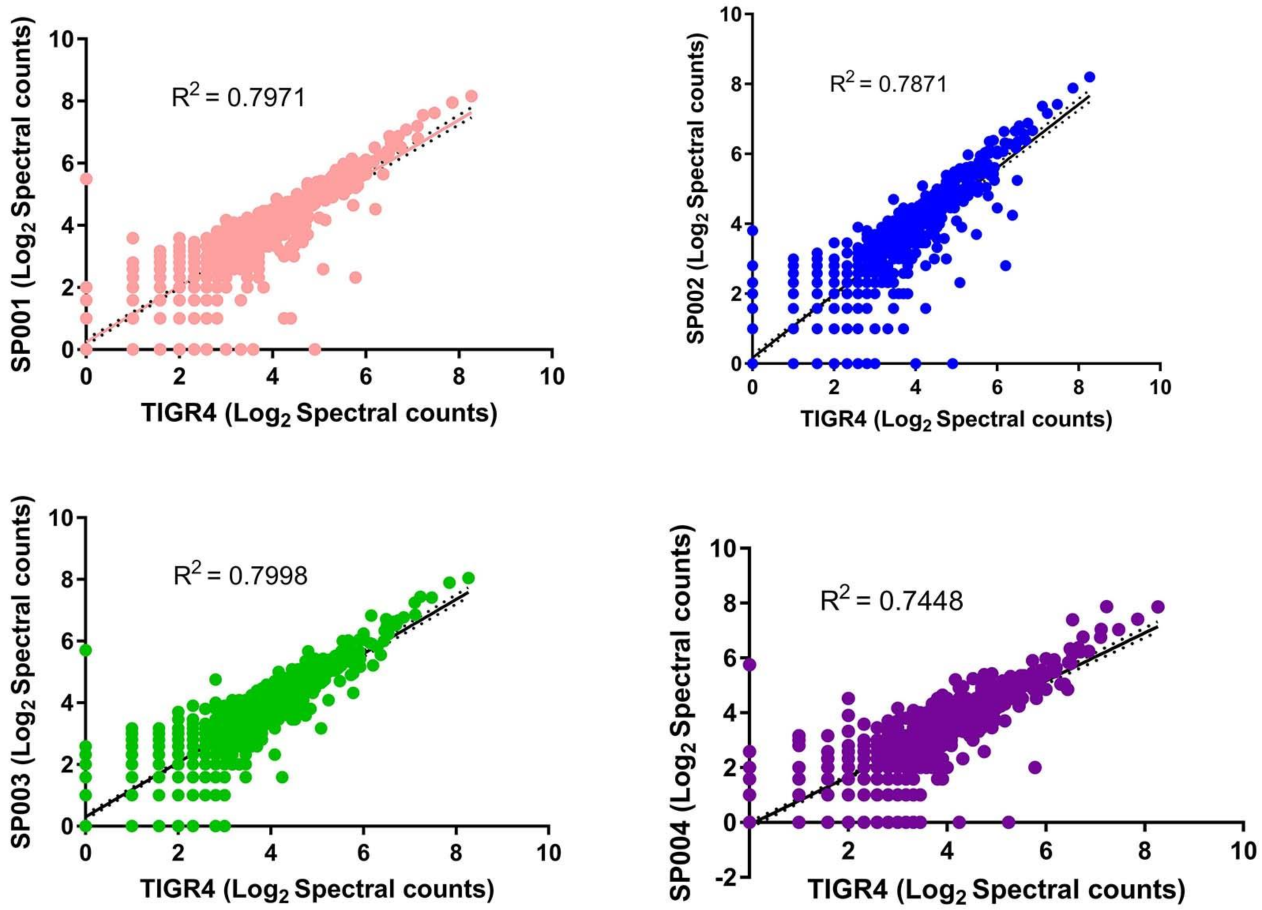

Supplementary figure 3. Related to figure 2. Similarity of NET-inducing clinical isolates of pneumococci to TIGR4. The $\log_2$ transformed spectral count values obtained from the clinical isolates SP001, SP002, SP003 and SP004, and TIGR4 were plotted. A linear regression analysis was performed. All clinical strains of pneumococci were found to contain proteins highly similar to the reference strain TIGR4.

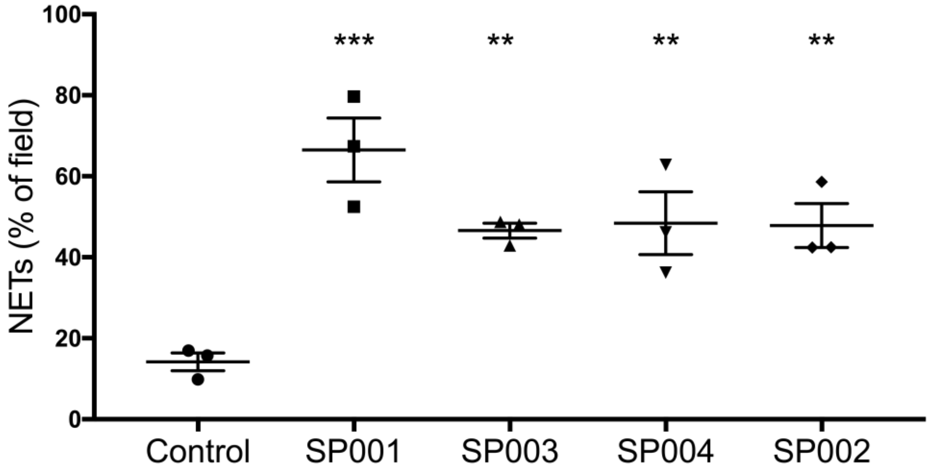

Supplementary figure 4. Related to Figure 2. NET formation by rat neutrophils

Neutrophils isolated from rats were stimulated with various strains of *S. pneumoniae* or saline control. NETs were visualized by immunofluorescence against rat myeloperoxidase and DNA and the relative amount of NETs was quantified using Fiji. All strains were compared to the control by one-way ANOVA followed by Sidak's multiple comparison test (*** $p<0.001$, ** $p<0.01$). Bars denote mean + standard deviation.

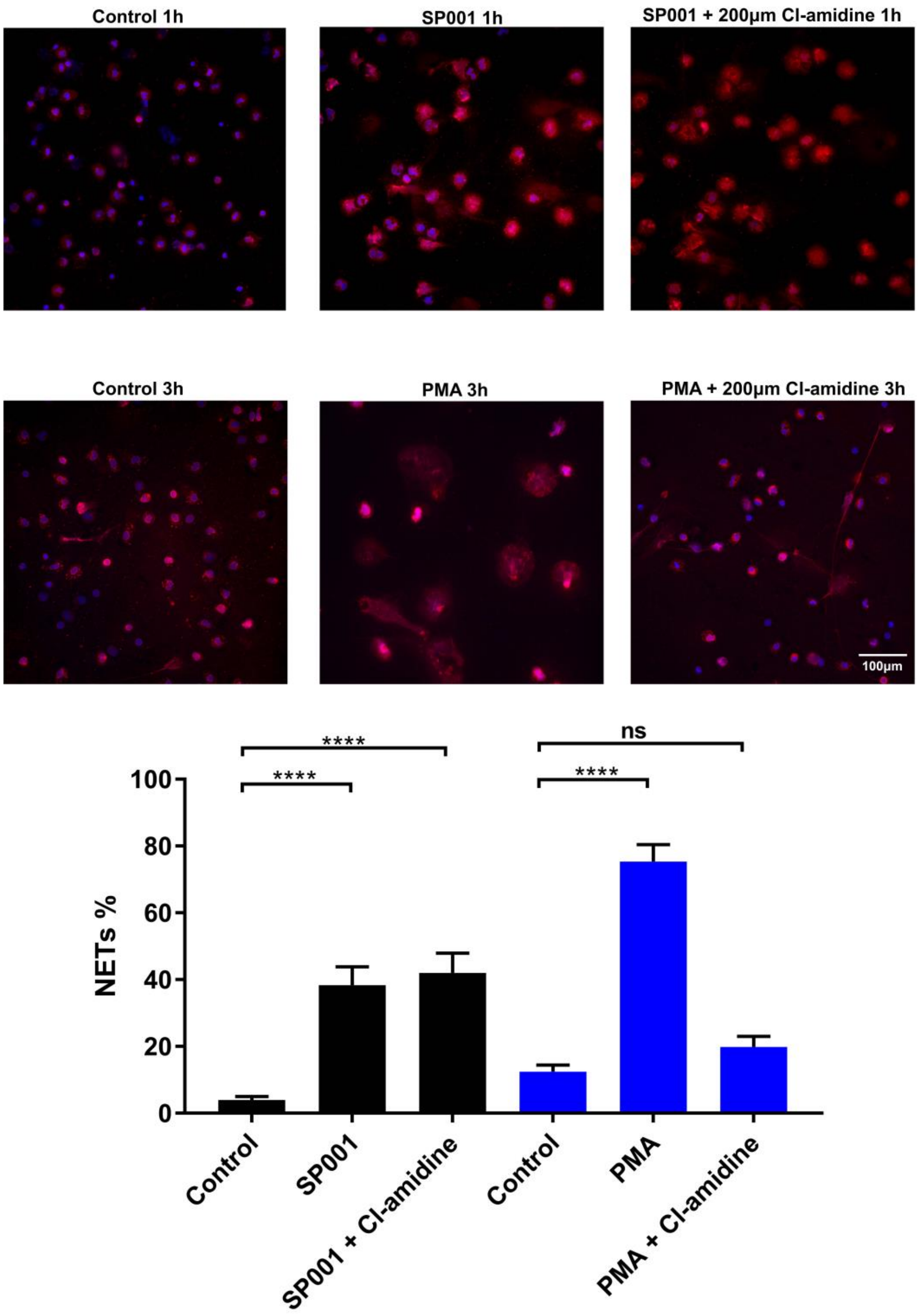

Supplemental figure 5 related to figure 2. Cl-amidine does not suppress NET induction by SP001.

Neutrophils isolated from the blood of healthy human donors were preincubated with 200µM Cl-amidine or without ant pre-treatment for 15 minutes prior to stimulation with pneumococcal strain SP001 for 1 hour or with PMA for 3 hours. NET induction was

quantified using Fiji and expressed as percentage of NETs. All conditions were compared to non-stimulated controls using one-way ANOVA followed by Sidak's multiple comparisons test (*** $p<0.001$, ** $p<0.01$, n.s – non-significant). Bars denote mean + standard deviation.

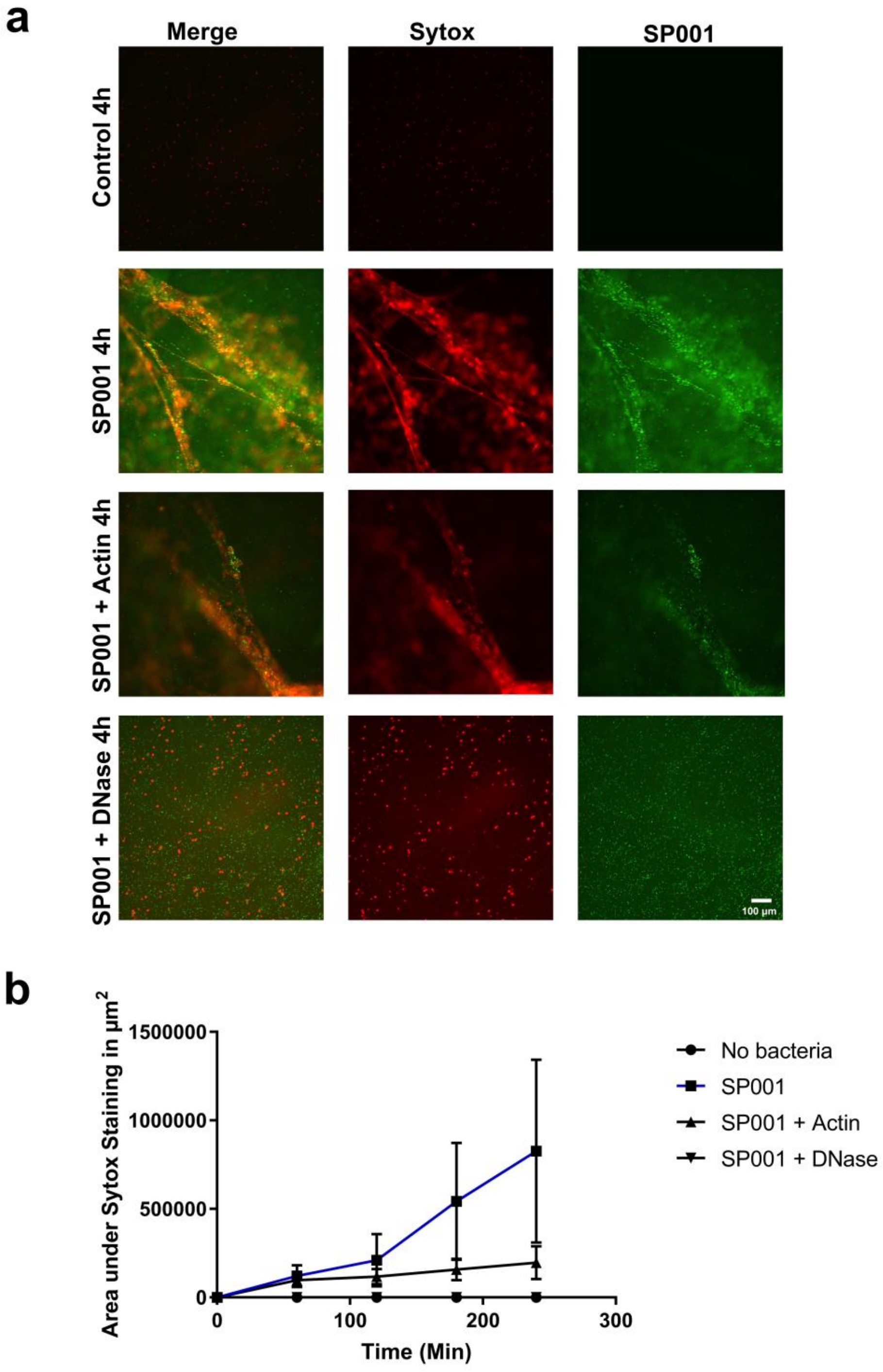

Supplemental figure 6. Related to figure 2. SP001 does not degrade NETs. NETs were induced with Oregon green labelled SP001 (green) in presence of actin or DNase (n =4). Images were acquired at 0', 60', 120' and 240'. Extracellular DNA was stained with a non-permeable dye Sytox orange (red). **a)** Representative images of NETs (red) and bacteria (green) after 4 hours of incubation. **b)** Area under Sytox staining was

quantified using Fiji. Thresholding was performed using the threshold function and Phansalkar setting. A Cut-off of 500 square microns was used to exclude Sytox-positive cytoplasts in DNase treated samples. Points represent average values and error bars denote standard deviation.

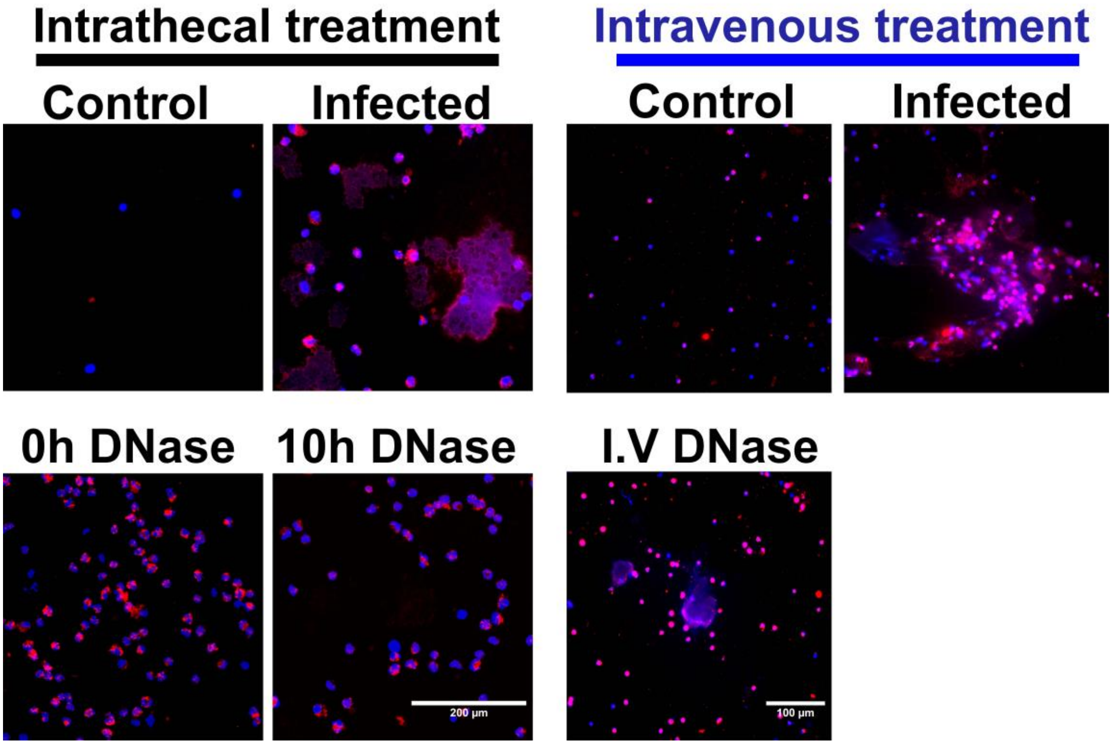

Supplementary Figure 7 related to figure 4 Dissolution of neutrophil extracellular traps (NETs) formed in a rat model of pneumococcal meningitis by intrathecal or intravenous administration. To determine the effect of intrathecal DNase treatment, infected rats either received a subarachnoid infusion of 10 units of DNase simultaneously (0h) or 10 hours (10h) after the infection, or they received an equal volume of saline vehicle solution simultaneously to the infection. To determine the effect of intravenous DNase treatment, infected rats either received an intravenous bolus dose of 3500 units of DNase 6 hours (6h) after the infection, followed by

intravenous infusion of 780 units/hour over the next 18 hours, or they received an equal volume of saline vehicle control in the same manner. In all cases, uninfected (control) rats received an equal volume of saline vehicle control either intrathecally or intravenously as indicated. All rats were sacrificed 24 hours after the infection. Cerebrospinal fluid was collected for visualization of NETs only by immunofluorescence against rat myeloperoxidase (red) and DNA (blue). Areas of red and blue co-localization represent NETs.

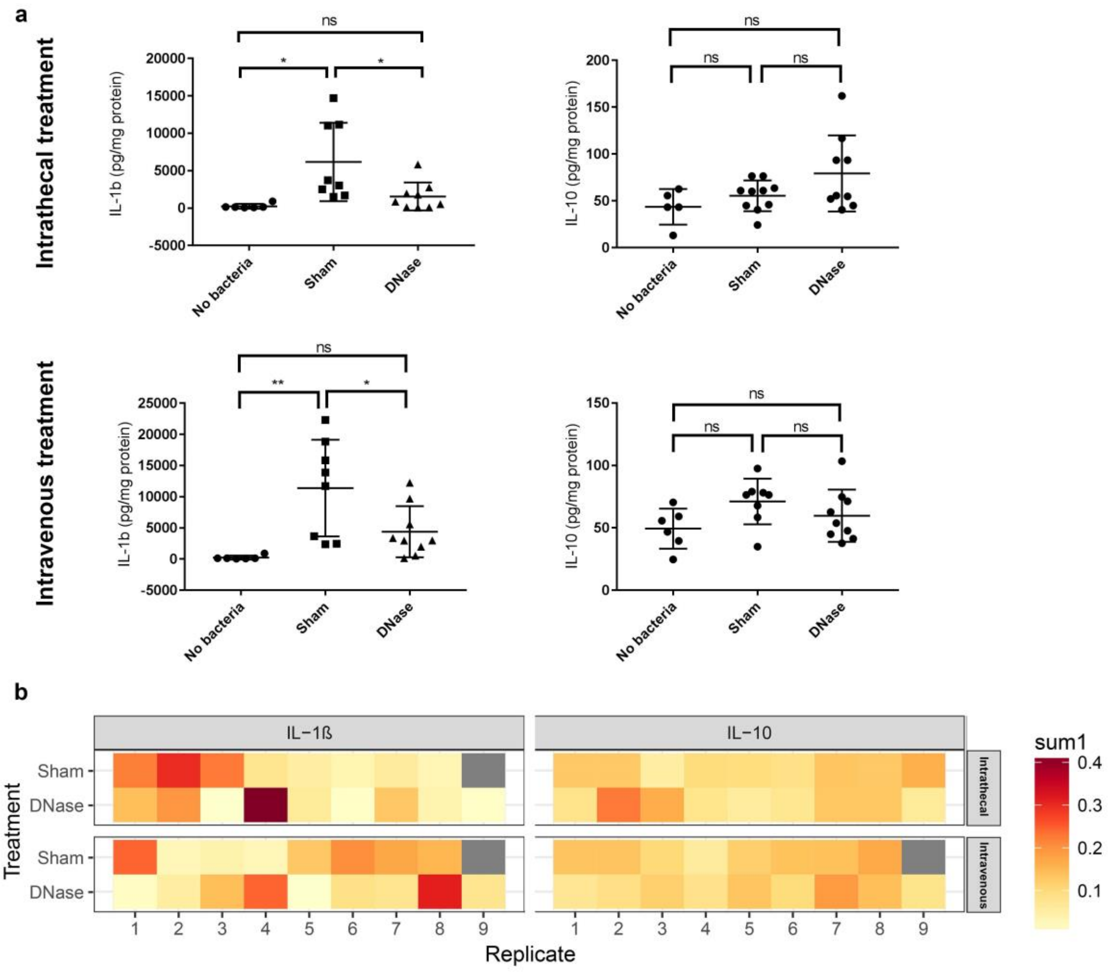

Supplementary figure 8 related to figure 4. DNase treatment reduces Il-1b in infected rats. To determine the effect of intrathecal DNase treatment, infected rats either received a subarachnoid infusion of 10 units of DNase simultaneously 0 hours after the infection, or they received an equal volume of saline vehicle solution (sham)

simultaneously to the infection. To determine the effect of intravenous DNase treatment, infected rats either received an intravenous bolus dose of 3500 units of DNase 6 hours (6h) after the infection, followed by intravenous infusion of 780 units/hour over the next 18 hours, or they received an equal volume of saline vehicle control in the same manner. In all cases, uninfected (control) rats received an equal volume of saline vehicle control either intrathecally or intravenously as indicated. All rats were sacrificed 24 hours after the infection. The rats were sacrificed and the brain was removed and immediately homogenized. **a)** Il-1b and Il-10 was measured in the whole brain homogenates. Groups were compared by one-way ANOVA followed by Sidak's multiple comparison test (*** $p<0.001$). Data depicts mean values and error bars denote standard deviation.

b) A heat map depicting individual Il-1b and Il-10 values from rats treated either intrathecally or intravenously is presented.

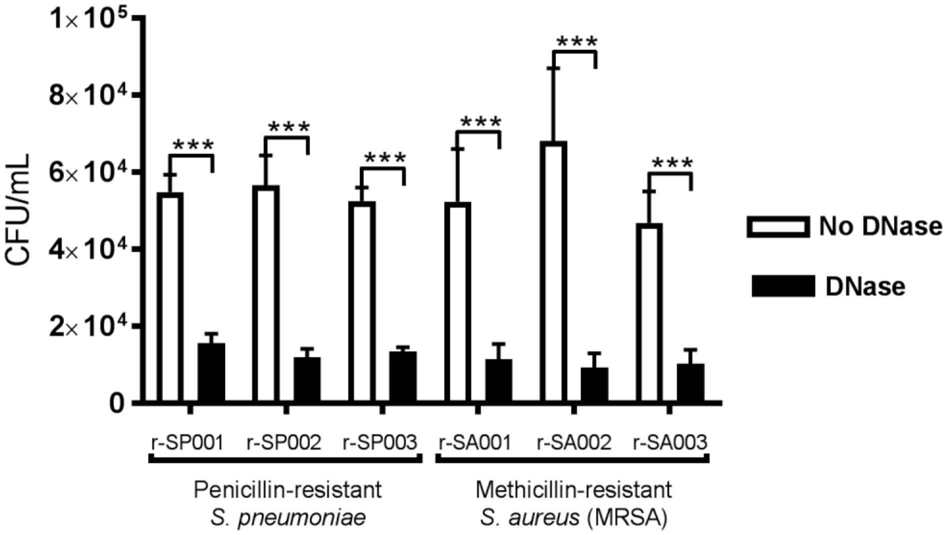

Supplementary figure 9. Related to Figure 3. DNase increases bacterial killing of antibiotic resistant strains of *S. pneumoniae* and MRSA. Neutrophils were challenged with several strains of either penicillin resistant *S. pneumoniae* or MRSA in the presence or absence of DNase. The resulting number of viable colony forming units (CFU) of bacteria was determined after 3 hours of stimulation. Indicated groups were compared by one-way ANOVA followed by Sidak's multiple comparison test (*** p<0.001). Bars denote mean values + standard deviation

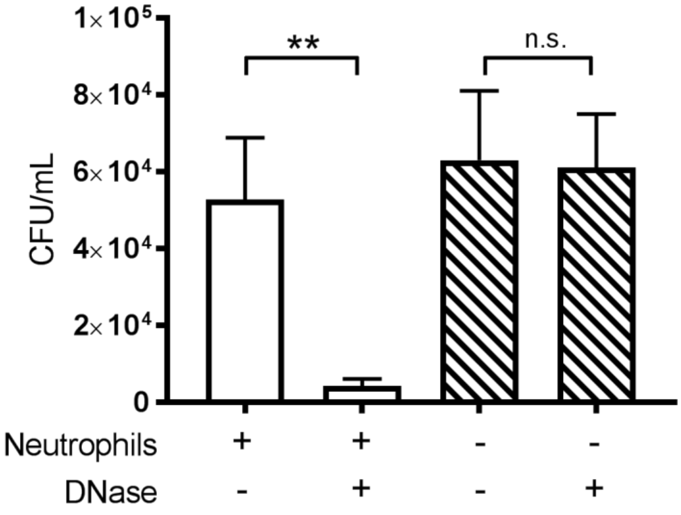

Supplementary figure 10. Related to Figure 5. The presence of neutrophils is required for DNase-enhanced killing of bacteria

The SP001 strain of *S. pneumoniae* was stimulated with or without DNase in the presence or absence of neutrophils. The number of viable colony forming units of bacteria was determined after 3 hours of stimulation. Indicated groups were compared by one-way ANOVA followed by Sidak's multiple comparison test (** p<0.01). Bars denote mean values + standard deviation

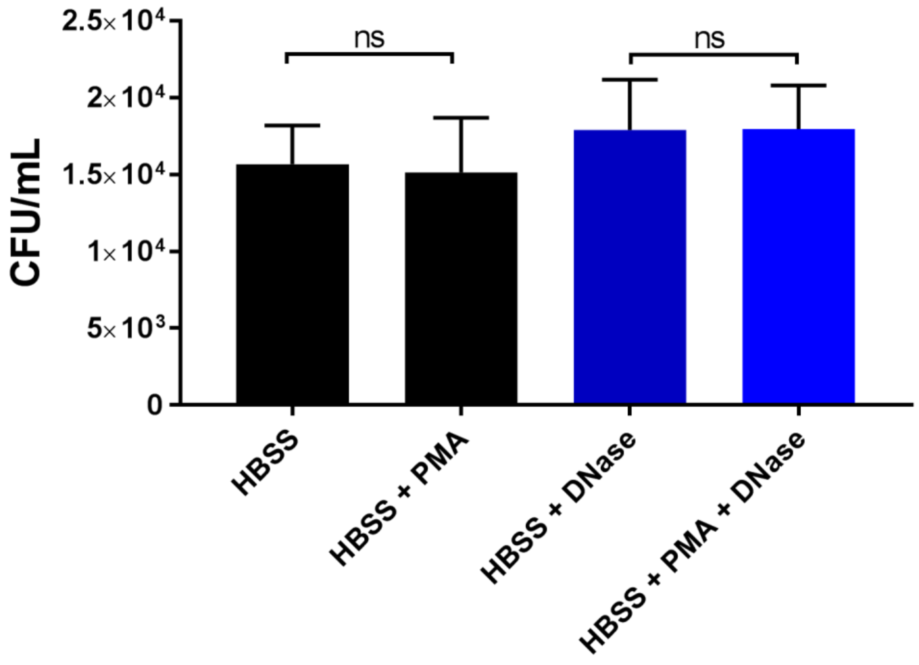

Supplementary figure 11. Related to figure 5. Concentrated DNase does not inhibit bacterial growth.

SP001 strain of *S. pneumoniae* was incubated with concentrates from control supernatants obtained simultaneously with DNase-treated NETs. The number of viable colonies was determined after 3 hours of incubation. Indicated groups were compared by one-way ANOVA followed by Sidak's multiple comparison test (n.s – non-significant). Bars denote mean values + standard deviation.

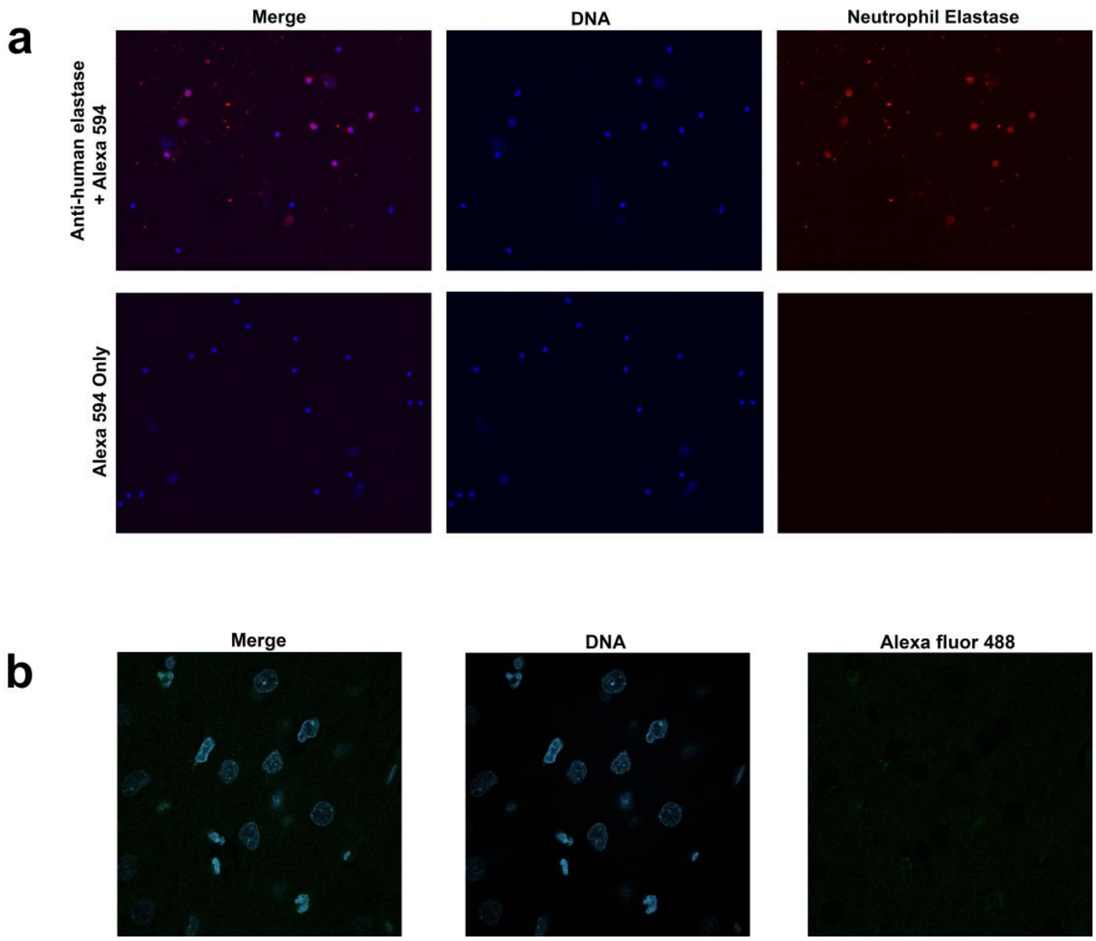

Supplement figure 12. Negative staining controls for Human CSF cytospins using Alexa594 and rat brains using Alexa488. a) Cytospins from a patient with subarachnoid hemorrhage were immunostained with primary rabbit anti-human neutrophil elastase and secondary goat anti-rabbit Alexa fluor 594 conjugated antibody or with secondary goat anti-rabbit Alexa fluor 594 conjugated antibody alone. **b)** Rat brain sections were stained with Alexa fluor 488 conjugated antibody alone.

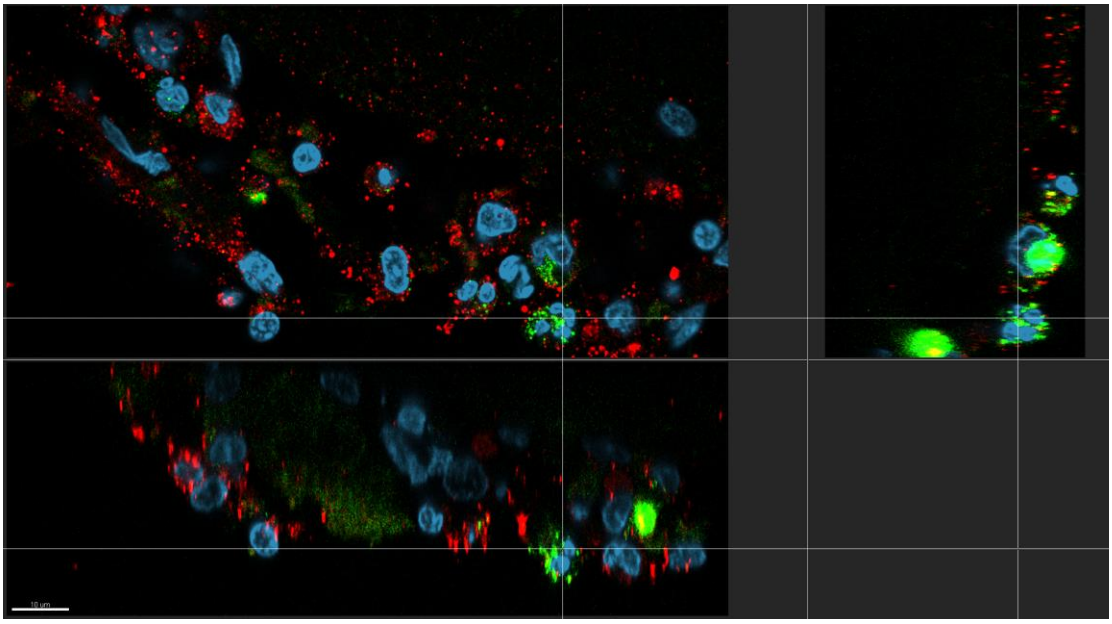

Supplementary figure 13. Bacteria remain unbound to NETs. Representative confocal images of infected rat brain tissue using immunofluorescence against SP001 (red), DNA (blue) and myeloperoxidase (green). Scale bar represents 10μm.

Methods

Patient sample collection

The study was approved by the Lund University ethics committee (Dnr 2016/672) and consent was also obtained from patients or next of kin. Cerebrospinal fluid (CSF) was obtained from patients with *S. pneumoniae* meningitis (n =6), neuroborreliosis (n = 3), acute viral meningitis (n = 4), and trauma (n = 3) by lumbar puncture or through an intraventricular device, if it was installed. CSF samples were kept at 4°C and were processed within 0-24 hours after collection.

Detection of NETs

CSF samples were fixed with an equal volume of 4% paraformaldehyde (Sigma-Aldrich) for 30 minutes at 4°C. The samples were then cytocentrifuged on to glass slides (Thermo Fisher). Samples were permeabilized with 0.5% triton-X-100 (Sigma-Aldrich) and blocked with 5% goat serum (BioWest). Some samples from ABM patients were also treated with either heparin (Leo, 1 Unit/mL), or DNase (Abcam, 5U/mL) for (20 min at 37°C) prior to fixing and cytocentrifugation.

Human samples were stained with rabbit-anti-human neutrophil elastase (Dako), and detected with Alexafluor 594-labelled secondary goat-anti-rabbit F_{ab} antibody fragment (Life Technologies). Rat samples were stained with anti-mouse myeloperoxidase, validated to cross react with rat myeloperoxidase[1] (Novusbio), and detected with Alexafluor 594-labelled secondary goat-anti-rabbit Fab antibody fragment (Life Technologies). Coverslips (#1, Menzel Glaser) were mounted on top of the samples with mounting media containing 4',6-diamidino-2-phenylindole (DAPI) (Life Technologies) and visualized with a Nikon Ti-E microscope. Images were acquired Andor Neo/Zyla camera and NIS elements advanced research software (Nikon).

Preparation of samples for shotgun mass spectrometry

CSF samples were centrifuged for 5 minutes at 350 RCF, which yielded a pellet of cells and/or NETs and a clear supernatant. The samples were subjected to trypsin digestion, C18 cleanup and analyzed by mass spectrometry (see below).

Double digestion of bacterial samples for shotgun mass spectrometry

Pneumococci were grown to mid-logarithmic phase, washed in PBS and pelleted down. Intracellular proteins were isolated from the bacteria using a previously published method[2]. Double digestion was performed on bacterial lysates as described earlier[3].

Preparation of NET supernatants for mass spectrometry

Neutrophils were isolated from healthy human donors using polymorphprep using polymorphprep (Axis-Shield) according to the manufacturer's directions and resuspended to the desired number in HBSS with calcium and magnesium (Life technologies). 1 x 10^6 neutrophils in 350µl HBSS containing calcium and magnesium were aliquoted in low-bind microfuge tubes (Eppendorf). NETs were induced by treating cells with 20nM PMA for 3 hours at 37°C. After 3 hours, 5U DNase was added to non-stimulated cells and PMA treated cells, and further incubated for 20 minutes at 37°C under rotation. Samples were spun at 300g for 8 minutes to pellet cells. The supernatant was removed and concentrated using 10 K centrifuge filters (Microcon, Millipore). The concentrate was resuspended in a final volume of 100µl 10 mM Tris and 140mM NaCl; pH =7.4 and protein estimation was performed using BCA kit (Pierce). 20µg of protein was taken for digestion and peptide clean-up as described below.

Protein digestion and peptide clean-up

Proteins were reduced with 5 mM dithiothreitol (Sigma, USA) for 45 min at 37 °C, and alkylated with 25 mM iodoacetamide (Sigma, USA) for 45 min followed by dilution with 100 mM ammonium bicarbonate to a final urea concentration below 1.5 M. Proteins were digested by incubation with trypsin (1/100, w/w) for at 18 hours at 37 °C. The peptides were cleaned up by C18 reversed-phase spin columns as per the manufacturer's instructions (Harvard Apparatus, USA).

LC-MS/MS analysis

All peptide analyses were performed on a Q Exactive Plus mass spectrometer (Thermo Fisher Scientific) connected to an EASY-nLC 1000 ultra-high-performance liquid chromatography system (Thermo Fisher Scientific). For data-dependent acquisition, peptides were separated on an EASY-Spray column (Thermo Scientific; ID 75 µm x 25 cm, column temperature 45 °C). Column equilibration and sample load were performed using constant pressure at 600 bars. Solvent A was used as stationary phase (0.1 % formic acid), and solvent B (mobile phase; 0.1 % formic acid, 100% acetonitrile) was used to run a linear gradient from 5 % to 35 % over 60 min at a flow rate of 300 nl/min. One full MS scan (resolution 70,000 @ 200 m/z; mass range 400-1,600 m/z) was followed by MS/MS scans (resolution 17,500 @ 200 m/z) of the 15 most abundant ion signals (TOP15). The precursor ions were isolated with 2 m/z isolation width and fragmented using higher-energy collisional-induced dissociation at a normalized collision energy of 30. Charge state screening was enabled and unassigned or singly charged ions were rejected. The dynamic exclusion window was set to 10 s. Only MS precursors that exceeded a threshold of 1.7e4 were allowed to trigger MS/MS scans.

The ion accumulation time (IT) was set to 100 ms (MS) and 60 ms (MS/MS) using an automatic gain control (AGC) target setting of 1e6 (MS and MS/MS). All data-independent acquisition (DIA) peptide analyses were performed on a Q Exactive HF-X mass spectrometer (Thermo Fisher Scientific) connected to an EASY-nLC 1200 ultra-high-performance liquid chromatography system (Thermo Fisher Scientific). For DIA acquisition, peptides were separated on an EASY-Spray column (Thermo Scientific; ID 75 µm x 50 cm, column temperature 45 °C). Column equilibration and sample load were performed using constant pressure at 900 bars. Column equilibration and sample load were performed using constant pressure at 900 bars. Solvent A was used as stationary phase (0.1 % formic acid), and solvent B (mobile phase; 0.1 % formic acid, 80% acetonitrile) was used to run a linear gradient from 5 % to 35 % over 60 min at a flow rate of 300 nl/min. One full MS scan (resolution 120,000 @ 200 m/z; mass range 350-1,650 m/z; IT 100 ms, AGC 3e6) was followed by 26 DIA scans with variable window sizes (inclusion list: 366.5, 395, 418, 438, 457, 475, 493, 511.5, 529.5, 547.5, 565.5, 584, 603.5, 623.5, 644.5, 666.5, 689, 713, 740.5, 771, 804.5, 842, 887.5, 946, 1027.5, 1363 m/z; isolation windows: 33, 26, 22, 20, 20, 18, 20, 19, 19, 19, 19, 20, 21, 21, 23, 23, 24, 26, 31, 32, 37, 40, 53, 66, 99, 574 m/z; resolution 30,000 @ 200 m/z; IT 50 ms, AGC 3e6).The obtained raw files were gzipped and Numpressed4 and converted to mzML format using msconvert from ProteoWizard. Data were stored and managed using openBIS[4].

Data analysis

The DDA spectra were searched against the human reference proteome acquired from UniProt (UP000005640, Oct-2015, reviewed and canonical proteins only) by using

Trans-Proteomic Pipeline (TPP v4.7 POLAR VORTEX rev 0, Build 201405161127) with X!Tandem. For decoy protein generation, a reverse approach was used. The following modifications were considered: fixed cysteine carbamidomethylation, variable methionine oxidation, variable acetylation of the n-terminae and variable S-carbamoylmethyl-cysteine cyclization of the n-terminal cysteines. Pyro-glutamic acid formation from glutamic acid and glutamine were allowed and enzyme specificity was set to allow two missed cleavage sites for trypsin. The mass tolerance thresholds were set to 20 ppm for precursors and 50 ppm for fragments. False discovery rates calculated using a target-decoy approach at protein, peptide and spectrum match level by the software tool Franklin[5] and set to <0.01. For protein abundance estimations, spectra of proteotypic peptides were summed for each identified protein (spectral counting).

DIA-Mass spectrometry

Computational workflows for analysing DIA data was executed and managed by openBIS[4] and included OpenSWATH v2.0[6] data extraction using a assay library of human purified neutrophils (unpublished: E. Malmström, S. Hauri, Johan Malmström), pyprophet-cli 0.0.19[7] for false discovery rate (FDR) estimation and the FDR was set to 1% at peptide precursor level and at 1% at protein level, and TRIC[8] for reducing the identification error. The quantitate protein matrix was filtered against NET-bound proteins[9] and the individual replicate protein intensities were normalized against respective total protein intensity per donor. Statistical tests and plotting of were done with ggpubr and the heatmap was drawn with complex Heatmap[10].

Induction of NETs *in vitro*

Neutrophils from humans and rats were isolated using polymorphprep (Axis-Shield) according to the manufacturer's directions and resuspended to the desired number in

HBSS with calcium and magnesium (Life technologies). Coverslips were washed with PBS and incubated in 24-well plates with 0.01 % poly-L-lysine (Sigma) in sterile PBS overnight at 37 °C. Coverslips were washed once in PBS and 200 µL of 3.5 x 10^5 PMNs/ml and bacteria (MOI 1:10) were added to each well. Samples were then fixed and immunostaining was performed on the samples as mentioned above. Neutrophils in HBSS (with calcium and magnesium) only were used as non-stimulated controls.

Quantification of NETs

For detection of NETs in patient CSF and animals, 3 to 5 random images at 20X or 10X magnification were used for quantifying each condition. NETs were defined as a localization of DNA and neutrophil elastase and expressed as % NETosis of field.

For in vitro NET formation, 3 to 5 random images at 20X or 10X magnification, with a minimum of 175 cells in total were used for quantification for each condition. The DAPI channel was used to identify nuclei. The elastase positive area in non-stimulated cells with normal polymorphonuclear morphology as described by Mohanty et al[11]. An increase in elastase-positive area of 33% was used as a cut-off to eliminate non-activated cells and detect NET formation in the samples. Image analysis was performed with the public domain software (Fiji).

Bacterial culture

Clinical isolates of various bacteria were collected from either blood or CSF from patients after being diagnosed with meningitis at the clinic for infectious diseases at Skåne University Hospital in Lund, Sweden.

For survival assays, animal infection and NET-induction, all *S. pneumoniae* strains were cultured overnight on blood agar (5% Sheep's blood) and then the colonies from

the entire plate were transferred into a total of 7mL of Todd-Hewitt medium with 0.5% yeast extract (BD Biosciences) supplemented with 10% choline chloride (Sigma). Upon reaching an optical density (OD) of 0.4 they were washed and resuspended in phosphate buffered saline solution (sigma) at an appropriate dilution for further experiments.

All *S. aureus* strains were cultured in tryptic soy broth (TSB) overnight, washed and used for NET induction. For survival assays, bacteria were cultured from an overnight culture until the bacteria reached mid log phase, washed with PBS and set to the desired OD.

All other bacteria were cultured overnight: *N. meningitidis* in GC broth with 10% FBS, L. monocytogenes in beef heart infusion (BHI), *A. baumannii* and *E. coli* in Luria bertani (LB), *S. capitis, S. oralis* and *S. epidermidis* in Todd-Hewitt medium with 0.5% yeast extract. They were then washed with PBS and resuspended to the desired OD. All media were purchased from BD biosciences.

<u>Rat model of meningitis</u>

The local Ethical Committee for Animal Research (M80-14) approved the experimental protocol. Adult male rats (Taconic, 350-370g) were used. Animals were treated in accordance with the National Institutes of Health for the Care and Use for Laboratory animals. Anaesthesia was induced with pentobarbital (30 mg/kg). Body weight was recorded and body temperature, measured rectally, was maintained at 37°C. All surfaces, instruments, and the head of the rat were cleaned with 70% ethanol before the procedure. The head was fixed on a stereotactic device and the skull was exposed. A hole was drilled in front of the lamboid suture and to the left of the sagittal suture using an automated hollow drill (5mm diameter). The piece of bone inside the drilled area was lifted and set aside. The durum and arachnoid membranes were carefully punctured

with a needle (27G) and a catheter (32G) was inserted into the subarachnoid space. Either 20µL of bacterial solution (3 x 10^6 bacteria), or the same bacterial solution containing 10 Units of recombinant human DNase I (Abcam), or sterile physiological saline solution (Fresenius Kabi), was injected into the subarachnoid space using a syringe pump at a flow rate of 2µL/min. After the infusion was finished, the cathether was left in place for 1-2 minutes more to minimize backflow upon removal of the catheter. The catheter was removed and the piece of bone was cleaned with ethanol and replaced to keep out unwanted bacteria and sealed with histoacryl (Braun). The incision was sealed and wiped with ethanol.

After 24 hours, anaesthesia was induced with pentobarbital as above. Weight was recorded and temperature measured rectally. The head was fixed in the stereotactic device and the incision reopened and the piece of bone removed to expose the meninges. A needle (27G) was inserted into the subarachnoid space and approximately 10µL of CSF was aspirated and diluted with 200µL of physiological saline. NETs were visualized in CSF samples as above.

Blood samples were collected via the left femoral artery. Rats were killed by decapitation and organs were removed and placed in saline (brain - 2ml, lungs and spleen – 800µL).

<u>10-hour DNase treatment in rats</u>

Recombinant human DNase I (Abcam) was infused subarachnoidally 10 hours after bacterial infection. In rats receiving treatment at 10 hours, anaesthesia was induced by isofluorane gas. The skull was re-exposed and the previously replaced piece of bone was again removed to expose the brain. A catheter was placed subarachnoidally as

described above and 10 Units of DNase was infused at a flow rate of 2µL/min. The piece of bone was replaced and sealed with histoacryl and the wound was again closed.

Intravenous DNase treatment in rats

Bacteria or saline vehicle control was infused subarachnoidally in rats as described above. For intravenous administration, a catheter was inserted into the jugular vein (outer diameter 1.19mm, Silastic) and secured to the back of the neck. Six hours after bacteria or vehicle infusion, a bolus dose of 3500 units of DNase (Worthington) or equal volume saline vehicle solution was infused intravenously using a syringe pump. Then a continuous infusion of 780 units/hour of DNase or equal volume saline vehicle solution at a flow rate of 0.05mL/hour was initiated. This infusion continued until the rats were sacrificed after 24 hours as described above.

Blood Brain Barrier (BBB) permeability

BBB permeability was assessed by measuring the blood to brain transfer constant (Ki) for ^{51}Cr-EDTA as described in detail previously[12]. Briefly, at about 23 h after injection of bacteria animals were reanaesthetized. A bolus of approximately 50 kBq of ^{51}Cr-EDTA (Nycomed Amersham, Stockholm, Sweden) was given intravenously, followed by an infusion at a rate of 200 kBq/h. Arterial blood samples (10 µL) were collected at every 2.5 to 5 minutes for 40 minutes. At 37 min, about 70 kBq ^{125}I-albumin was given intravenously. At 40 minutes the animals were decapitated. A 8-mm coronal section of the cortex centered over the insertion point for bacteria was removed and weighed. ^{51}Cr and ^{125}I activities in tissue and blood samples were determined in a gamma counter. Arterial hematocrit was measured at the start and at the end of the tracer infusion, and was used to convert blood concentrations into plasma concentrations. Ki was then

calculated as: Ki = B/ $_0\int^T$ Ca (t) dt where B is the amount of tracer in the tissue, Ca is the concentration of the tracer in arterial plasma as a function of time, and T is the duration of the experiment. B was calculated as the total ^{51}Cr activity in the tissue samples minus the ^{51}Cr activity in the cortical plasma volume determined by the distribution volume for ^{125}I-albumin in the tissue sample.

Bacterial counts in rat organ homogenates

The brain, lungs and spleen were homogenized using silicone beads in a TissueLyser (Qiagen) and 20μL of fresh homogenate was plated onto blood agar plates and incubated at 37°C overnight. Colony forming units were counted and confirmed to be S. pneumoniae based on colony characteristics. The rest of the homogenate was flash frozen in liquid nitrogen for 10 minutes and then stored at -80°C.

IL-6 in brain homogenate

Brain homogenate was mixed with T-PER lysis buffer (ThermoFisher) according to manufacturer's directions. Protein content in the lysate was measured by bicinchoninic acid (BCA) protein assay kit (Thermo Fisher). IL-6 was measured in the lysed homogenate using a Quantikine rat IL-6 ELISA kit (R&D Systems) according to manufacturer's directions. IL-6 levels were normalized to the amount of protein in each sample, expressed as pg IL-6 per mg of protein.

Bacterial killing after DNase treatment

NETs were induced in human neutrophils as above, with the simultaneous addition of DNase (5 Units) or equal volume of saline solution. To determine the killing mechanism, the following inhibitors were added 30minutes after addition of DNase and

bacteria: 10µM Cytochalasin D (Calbiochem), 10µM myeloperoxidase inhibitor 4-Aminobenzoic hydrazide (4-ABAH) (Calbiochem) or 10µM NADPH oxidase inhibitor Diphenyl iodonium chloride (DPI) (Calbiochem). Samples were incubated for 2.5 hours after addition of inhibitors. NETs were detected as above using immunofluorescence. Samples were then diluted and 20µL of a ten-fold dilution of each sample was streaked on blood agar plates and colony-forming units of S. pneumoniae were determined as above.

Myeloperoxidase (MPO) activity assay

After 30 minutes of in vitro NET induction in the presence and absence of DNase as described above, samples were centrifuged and the cell-free supernatant analyzed for MPO peroxidation and chlorination activity using EnzChek MPO Assay Kit (Molecular Probes) according to the manufacturer's directions. Due to the high background peroxidise activity of plasma, it was excluded from all stimulation conditions. We verified that bacterial killing in the presence and absence of DNase occurred to a similar extent under these conditions.

Gentamicin assay

SP001 was cultured in THY medium supplemented with choline chloride as described previously. The bacteria were then added at MOI of 10 to 1 x 10^6 neutrophils in HBSS with 10% plasma alone, or in presence of DNase (5U) for 15 minutes at 37 degrees with shaking. Gentamicin (10µg/mL) was added was added for 30 minutes at 37 degrees to kill extracellular bacteria. Cells were washed thrice with HBSS and lysed with sterile water. The bacteria were then plated out at 10X or 100 dilutions and CFUs were counted to assess survival.

Visualization of phagocytosis

SP001 strain was labelled with Oregon green 488-X succinimidyl ester (20µM, Life Technologies) in PBS for 30 minutes in the dark at room temperature. The labelled bacteria were then washed thrice in PBS to remove excess unbound dye. Bacteria were then added to neutrophils in HBSS with 10% autologous plasma (MOI 1:10) alone or in the presence or DNase (5U) for on shaking for 1 hour at 37 degrees in the dark. Samples were then fixed, cytocentrifuged onto slides and processed for immunocytochemistry as described previously. Neutrophils were probed with rabbit human anti-elastase and DNA was visualized with DAPI.

Inhibition of NET formation by CL-amidine

Neutrophils were isolated from healthy human donors using polymorphprep (Axis-Shield) were seeded in 24 well plates on coverslips coated with 0.01% poly-lysine at a concetration of 7.5 x 10^5 cells/ml. Prior to stimulation with mid-log phase SP001(MOI1:10) or 20 nM PMA, cells were pre-treated with 200µM Cl-amidine (Calbiochem) for 20 minutes. Neutrophils were stimulated for 1 hour with SP001 or for 3 hours with 20 nM before fixation with 4% PFA. Samples were prepared for immunofluorescence using anti-human neutrophil elastase as described earlier. NETs were quantified using Fiji.

NET degradation assays using Sytox orange

Neutrophils were isolated from healthy human donors as mentioned earlier and were seeded in 8-well chamber plastic bottom microscopy slides (Ibidi) at concentration of 5 x 10^5 cells/ml. Mid-log phase SP001 labelled with Oregon green as mentioned earlier

and added (life technologies) at a MOI of 10 were to the wells containing neutrophils alone, in presence of 100µg/ml G-actin (New England Biolabs) or 1U of recombinant human DNase (Abcam). Sytox orange (Life technologies) was added to the wells at a dilution of 1:1000. Images were acquired at 0, 60, 120, 180 and 240 minutes.

References

1. Ge, L., *et al.* Neutrophil extracellular traps in ischemia-reperfusion injury-induced myocardial no-reflow: therapeutic potential of DNase-based reperfusion strategy. *Am J Physiol Heart Circ Physiol* **308**, H500-509 (2015).
2. Malmstrom, J., *et al.* Streptococcus pyogenes in human plasma: adaptive mechanisms analyzed by mass spectrometry-based proteomics. *J Biol Chem* **287**, 1415-1425 (2012).
3. Sjoholm, K., Karlsson, C., Linder, A. & Malmstrom, J. A comprehensive analysis of the Streptococcus pyogenes and human plasma protein interaction network. *Mol Biosyst* **10**, 1698-1708 (2014).
4. Bauch, A., *et al.* openBIS: a flexible framework for managing and analyzing complex data in biology research. *BMC Bioinformatics* **12**, 468 (2011).
5. Teleman, J., Hauri, S. & Malmstrom, J. Improvements in Mass Spectrometry Assay Library Generation for Targeted Proteomics. *J Proteome Res* **16**, 2384-2392 (2017).
6. Rost, H.L., *et al.* OpenSWATH enables automated, targeted analysis of data-independent acquisition MS data. *Nat Biotechnol* **32**, 219-223 (2014).
7. Rosenberger, G., *et al.* Statistical control of peptide and protein error rates in large-scale targeted data-independent acquisition analyses. *Nat Methods* **14**, 921-927 (2017).
8. Rost, H.L., *et al.* TRIC: an automated alignment strategy for reproducible protein quantification in targeted proteomics. *Nat Methods* **13**, 777-783 (2016).
9. Urban, C.F., *et al.* Neutrophil extracellular traps contain calprotectin, a cytosolic protein complex involved in host defense against Candida albicans. *PLoS Pathog* **5**, e1000639 (2009).
10. Gu, Z., Eils, R. & Schlesner, M. Complex heatmaps reveal patterns and correlations in multidimensional genomic data. *Bioinformatics* **32**, 2847-2849 (2016).
11. Mohanty, T., *et al.* A novel mechanism for NETosis provides antimicrobial defense at the oral mucosa. *Blood* **126**, 2128-2137 (2015).
12. Bentzer, P., Venturoli, D., Carlsson, O. & Grande, P.O. Low-dose prostacyclin improves cortical perfusion following experimental brain injury in the rat. *J Neurotrauma* **20**, 447-461 (2003).
