# Supplementary material for: Neutrophil extracellular traps in the central nervous system hinder bacterial clearance during pneumococcal meningitis
Source: Nat Commun. 2019 Apr 10;10:1667. doi: 10.1038/s41467-019-09040-0 (PMC6458182; doi:10.1038/s41467-019-09040-0)
Supplement: Supplementary file 2 — Description of Additional Supplementary Files [file 41467_2019_9040_MOESM2_ESM.pdf]

Description of Additional Supplementary Files

File Name: Supplementary Data 1
Description: 1215 proteins were identified after LC-MS/MS analysis at 1 % FDR when searching the searching against the TIGR4 library (Swiss-Uniprot ID - UP000000585) . The table shows protein identification, uniprot accession number and spectral counts derived from pneumococcal strains. The spectral count data is presented in a raw non-normalized format.
