# Supplementary material for: Neutrophil extracellular traps in the central nervous system hinder bacterial clearance during pneumococcal meningitis
Source: Nat Commun. 2019 Apr 10;10:1667. doi: 10.1038/s41467-019-09040-0 (PMC6458182; doi:10.1038/s41467-019-09040-0)
Supplement: Supplementary file 4 — Reporting Summary [file 41467_2019_9040_MOESM4_ESM.pdf]

nature research

Corresponding author(s): Tirthankar Mohanty



# Reporting Summary

Nature Research wishes to improve the reproducibility of the work that we publish. This form provides structure for consistency and transparency in reporting. For further information on Nature Research policies, see Authors & Referees and the Editorial Policy Checklist.

## Statistical parameters

When statistical analyses are reported, confirm that the following items are present in the relevant location (e.g. figure legend, table legend, main text, or Methods section).

| n/a | Confirmed | |
|---|---|---|
| ☐ | ☒ | The exact sample size ($n$) for each experimental group/condition, given as a discrete number and unit of measurement |
| ☐ | ☒ | An indication of whether measurements were taken from distinct samples or whether the same sample was measured repeatedly |
| ☐ | ☒ | The statistical test(s) used AND whether they are one- or two-sided<br>*Only common tests should be described solely by name; describe more complex techniques in the Methods section.* |
| ☒ | ☐ | A description of all covariates tested |
| ☐ | ☒ | A description of any assumptions or corrections, such as tests of normality and adjustment for multiple comparisons |
| ☒ | ☐ | A full description of the statistics including central tendency (e.g. means) or other basic estimates (e.g. regression coefficient) AND variation (e.g. standard deviation) or associated estimates of uncertainty (e.g. confidence intervals) |
| ☐ | ☒ | For null hypothesis testing, the test statistic (e.g. $F$, $t$, $r$) with confidence intervals, effect sizes, degrees of freedom and $P$ value noted<br>*Give P values as exact values whenever suitable.* |
| ☒ | ☐ | For Bayesian analysis, information on the choice of priors and Markov chain Monte Carlo settings |
| ☒ | ☐ | For hierarchical and complex designs, identification of the appropriate level for tests and full reporting of outcomes |
| ☐ | ☒ | Estimates of effect sizes (e.g. Cohen's $d$, Pearson's $r$), indicating how they were calculated |
| ☐ | ☒ | Clearly defined error bars<br>*State explicitly what error bars represent (e.g. SD, SE, CI)* |

*Our web collection on statistics for biologists may be useful.*

## Software and code

Policy information about availability of computer code

| | |
|---|---|
| Data collection | No special data collection software was used for the study. |
| Data analysis | Data were analyzed using GraphPad Prism 7, R v3.2.3 as descibed in the methods section of the manuscript. |

For manuscripts utilizing custom algorithms or software that are central to the research but not yet described in published literature, software must be made available to editors/reviewers upon request. We strongly encourage code deposition in a community repository (e.g. GitHub). See the Nature Research guidelines for submitting code & software for further information.

## Data

Policy information about availability of data

All manuscripts must include a data availability statement. This statement should provide the following information, where applicable:

- Accession codes, unique identifiers, or web links for publicly available datasets
- A list of figures that have associated raw data
- A description of any restrictions on data availability

MS data have been deposited to ProteomeXchange via MassIVE partner repository with accession codes PXD011546. A reporting summary for this article has been included as a supplementary information file. All other data supporting the findings will be made available upon reasonable request from the corresponding author.

# Field-specific reporting

Please select the best fit for your research. If you are not sure, read the appropriate sections before making your selection.

☒ Life sciences ☐ Behavioural & social sciences ☐ Ecological, evolutionary & environmental sciences

For a reference copy of the document with all sections, see nature.com/authors/policies/ReportingSummary-flat.pdf

# Life sciences study design

All studies must disclose on these points even when the disclosure is negative.

| | |
|---|---|
| Sample size | No statistical methods/software were utilized to pre-determine sample size. The sample size was chosen based on high power and low variance to detect specific effects. |
| Data exclusions | No data were excluded from the study. |
| Replication | Results described in the manuscript have been replicated in independent experiments from multiple patients/donors. |
| Randomization | For preventing bias, all rats included in the intravenous treatments with DNase were randomly allocated to various groups. |
| Blinding | All intravenous treatments were performed in a blinded and randomized fashion. The correspondence between code and treatment was revealed only after results were obtained. |

# Reporting for specific materials, systems and methods

## Materials & experimental systems

| n/a | Involved in the study | |
|---|---|---|
| ☐ | ☒ | Unique biological materials |
| ☐ | ☒ | Antibodies |
| ☒ | ☐ | Eukaryotic cell lines |
| ☒ | ☐ | Palaeontology |
| ☐ | ☒ | Animals and other organisms |
| ☐ | ☒ | Human research participants |

## Methods

| n/a | Involved in the study | |
|---|---|---|
| ☒ | ☐ | ChIP-seq |
| ☒ | ☐ | Flow cytometry |
| ☒ | ☐ | MRI-based neuroimaging |

## Unique biological materials

Policy information about availability of materials

| | |
|---|---|
| Obtaining unique materials | All materials are available from commercial sources or upon request by the authors, except for cerebrospinal fluid and primary neutrophils. |

## Antibodies

| | |
|---|---|
| Antibodies used | Human CSF cytospins and in vitro stimulation of primary human neutrophils were stained with rabbit-anti-human neutrophil elastase (Dako, rabbit 1373 contract immunization, Dilution 1:500) and detected with Alexafluor 594-labelled secondary goat-anti-rabbit Fab antibody fragment (Life Technologies, Dilution 1:1000). Rat cytospin samples and in vitro stimulation of primary rat neutrophils were stained with anti-mouse myeloperoxidase 67 (Novusbio, NBP1-51148, Dilution 1:500) and detected with Alexafluor 594-labelled secondary goat-anti-rabbit Fab antibody fragment (Life Technologies, Dilution 1:1000).<br><br>Primary antibodies used for immunohistochemistry of rat brain sections were: chicken IgY anti-glial fibrillary acidic protein (GFAP; 1:500; PA1-10004, Thermo Fisher Scientific), mouse IgG1 anti-myeloperoxidase (MPO, 1:100; NBP1-51148, Novusbio) and rabbit anti-Streptococcus pneumoniae (SP; 1:100; PA1-7259, Thermo Fisher Scientific). Secondary antibodies conjugated to fluorophores used for staining were: (Alexa Fluor; 1:500; goat anti-mouse IgG1 AF568, goat anti-rabbit AF488, goat anti-rabbit AF568, goat anti-rat IgG2a AF488, goat anti-chicken AF568, Thermo Fisher Scientific) |
| Validation | Validation of antibodies are provided on the manufacturer's website. |

## Animals and other organisms

Policy information about studies involving animals; ARRIVE guidelines recommended for reporting animal research

| | |
|---|---|
| Laboratory animals | Adult male Sprague Dawley® rats (Taconic, 350-370g) were used. Animals were treated in accordance with the National Institutes of Health for the Care and Use for Laboratory animals. The local Ethical Committee at Lund University for Animal Research (M80-14) approved the experimental protocol. |
| Wild animals | No wild animals were used in the study. |
| Field-collected samples | The study did not inlvolve any samples collected from the field. |

## Human research participants

Policy information about studies involving human research participants

| | |
|---|---|
| Population characteristics | Cerebrospinal fluid (CSF) was obtained from patients with S. pneumoniae meningitis (3 males and 3 females), neuroborreliosis (2 males and 1 female), acute viral meningitis (2 males and 1 female), and subarachnoid hemorrhage (1 male and 3 females) by lumbar puncture or through an intraventricular device, if it was installed. The mean ± SD age for pneumococcal meningitis was 46 ± 24, for viral meningitis was 29 ± 13, for neuroborreliosis was 57 ± 10 and subarachnoid hemorrhage was 48 ± 23. Antibiotic treatment was initiated in patients with pneumococcal ABM between 0-96 hours from the time of CSF collection. |
| Recruitment | The study was approved by the Lund University ethics committee (Dnr 2016/672) and consent was also obtained from patients or next of kin. |
